# Modeling the Cost-Effectiveness of the Integrated Disease Surveillance and Response (IDSR) System: Meningitis in Burkina Faso

**DOI:** 10.1371/journal.pone.0013044

**Published:** 2010-09-28

**Authors:** Zana C. Somda, Helen N. Perry, Nancy R. Messonnier, Mamadou H. Djingarey, Salimata Ouedraogo Ki, Martin I. Meltzer

**Affiliations:** 1 Centers for Disease Control and Prevention, Atlanta, Georgia, United States of America; 2 WHO –IST West Africa, Ouagadougou, Burkina Faso; 3 Ministère de la Santé, Ouagadougou, Burkina Faso; Kenya Medical Research Institute, Kenya

## Abstract

**Background:**

Effective surveillance for infectious diseases is an essential component of public health. There are few studies estimating the cost-effectiveness of starting or improving disease surveillance. We present a cost-effectiveness analysis the Integrated Disease Surveillance and Response (IDSR) strategy in Africa.

**Methodology/Principal Findings:**

To assess the impact of the IDSR in Africa, we used pre- and post- IDSR meningococcal meningitis surveillance data from Burkina Faso (1996–2002 and 2003–2007). IDSR implementation was correlated with a median reduction of 2 weeks to peak of outbreaks (25^th^ percentile 1 week; 75^th^ percentile 4 weeks). IDSR was also correlated with a reduction of 43 meningitis cases per 100,000 (25^th^–40: 75^th^-129). Assuming the correlations between reductions in time to peak of outbreaks and cases are related, the cost-effectiveness of IDSR was $23 per case averted (25^th^-$30; 75^th^ - cost saving), and $98 per meningitis-related death averted (25^th^-$140: 75^th^ – cost saving).

**Conclusions/Significance:**

We cannot absolutely claim that the measured differences were due to IDSR. We believe, however, that it is reasonable to claim that IDSR can improve the cost-effectiveness of public health surveillance.

## Introduction

More than 1.5 million children die each year in sub-Saharan Africa, from diarrhea, malaria, measles, meningitis, respiratory infections, yellow fever, and HIV/AIDS [1]–[6]. Well known and effective interventions are available for controlling and preventing the diseases that cause these deaths but they are often not applied to their maximum potential [7]–[10]. The resulting deaths and the associated economic costs to society could be reduced if timely detection and control measures are implemented [11]–[13]. In response to this problem, in 1998, countries in the World Health Organization (WHO) African region adopted a regional strategy named Integrated Disease Surveillance and Response (IDSR) [14–16, Table S1]. IDSR is a strategy that seeks to strengthen the ability of national and regional public health surveillance programs. The goal of IDSR is to integrate a number of surveillance systems, both existing and newly formed. This integration should encompass all levels of public health (from the basic district-level through to the national level), and should achieve efficiencies by avoiding duplication of efforts. Areas of activity that IDSR focuses on to improve efficiency include detection and identification of public health problems, increased speed of reporting and notification (especially for immediately notifiable diseases), analysis of data and interpretation of trends, laboratory confirmation when required, decision-making about responses, monitoring of progress and regular evaluation of the surveillance system's quality (14–16). The net results of IDSR-implemented reforms in surveillance systems should be that outbreaks are detected earlier, allowing quicker public health response (e.g., vaccination campaigns).

Although considerable progress had been achieved with implementation of the IDSR strategy (see http://www.cdc.gov/idsr/implementation.htm#progress), the associated economic benefits (e.g., cases and death prevented, costs of medical treatments saved by the society, and the value of avoided year of life lost) are poorly documented. Most studies on economic evaluation of public health intervention programs in sub-Saharan Africa have focused on individual disease-specific intervention activities [17]–[23]. Relatively few studies have looked at the economic benefits of surveillance and response activities [24], [25]. In a previous study, we analyzed the costs of establishing and subsequently operating activities for detection and response to the priority diseases under the IDSR strategy [26]. We add to the literature by presenting a cost effectiveness analysis of IDSR, in which we will assume that any average reductions in health outcomes (e.g., incidence of cases and deaths, outbreak duration) were due to implementation of IDSR.

## Methods

To model the cost-effectiveness of IDSR, we used data from Burkina Faso because that country had fully established IDSR leadership and structures at the national level by 2002, with implementation at regional and district levels in 2003. Burkina Faso had data, collected using the IDSR-supported surveillance systems, on several meningitis outbreaks.

The nature of disease surveillance systems makes it impossible to have a randomly controlled experiment to measure the impact of IDSR on public health outcomes. We were unable to readily collect comparable data from another country (e.g., one without IDSR systems, or one that implemented IDSR systems after Burkina Faso), and thus we were unable to conduct a comparison between countries. We therefore relied on observational (before-and-after) data from outbreaks of meningococcal meningitis to assess the possible impact of IDSR-related activities in Burkina Faso. We assumed that any correlations between the start of IDSR activities, which includes both surveillance and response to disease activity detected, and changes in the epidemiology of meningitis outbreaks were due primarily to IDSR. With this assumption, we calculated, on an outbreak basis, costs per case, per death and per *sequelae* prevented. There could be other reasons for any correlations that we measured (see discussion section).

As most health care and IDSR activities in Burkina Faso are funded by the government, we took the perspective of the government-funded public health care system (i.e., we only recorded costs and savings incurred by the national government); costs incurred by households were not included. All cost data were recorded in local currency values and then converted into US dollar values using the mean annual exchange rate. We used the general consumer price index from Burkina Faso [27] and a discount rate of 3% to adjust all costs into 2002 US dollars equivalent.

### Epidemiological data

We obtained from the WHO Multi-Diseases Surveillance Center in Ouagadougou annual population data and district level reports of weekly meningitis cases and deaths from Burkina Faso for the years 1996–2007 (see Table S2). We then calculated the weekly incidence and mortality, expressed as cases and deaths per 100,000 inhabitants, by dividing the number of new cases and deaths occurring per week by the mean annual population of each reporting district. A 1988 study in The Gambia found that 27 out of 154 (17.5%) survivors of bacterial meningitis had generalized neurological sequelae [28] We therefore assumed 20% of meningitis survivors would have neurological defects (sequelae).

We sorted the data into two groups: before (1996–2002) and after (2003–2007) IDSR implementation at district level. During this study period, all meningitis outbreaks in Burkina Faso only occurred between January and June (23-week period). For each group, we examined the weekly incidence rates in relation to the WHO recommended alert threshold (5 cases per 100,000) and epidemic threshold (10 cases per 100,000) [29]. We defined the start (end)of an outbreak when cases in a district exceeded (returned below) the epidemic threshold. For each outbreak, we calculated the time-to-peak of the outbreak as the number of weeks elapsed from the first alert threshold to the week with the maximum weekly incidence (i.e., the peak of the outbreak). We also calculated the time to reach the median, 25^th^ and 75^th^ percentiles of cumulative total incidence and mortality.

For each group of outbreaks before and after IDSR implementation (start 2003), we calculated the median, 25^th^ and 75^th^ percentile for each of the following health outcomes: weekly and cumulative total incidence, mortality and sequelae.

### Data analysis

We first plotted the average weekly incidence rates over the time period studied and the median weekly incidence and mortality before and after IDSR implementation over the 23-week period of meningitis outbreaks. We then compared the health outcomes (i.e., incidence, mortality, time to peak and time to reach a set percentile of total cases per outbreak) using the Mann-Whitney test using SAS statistical software version 9.1 (SAS Institute Inc., Cary, NC, USA). In 1996 there was an “unusually” large epidemic of meningitis in Burkina Faso. We therefore examined the influence of 1996 data on the IDSR effectiveness measures by re-running the analyses excluding 1996 data.

### Response to outbreaks: Vaccine importation

As IDSR encompasses a deliberate response factor, it is plausible that vaccine imports may increase post-IDSR implementation. In order to assess potential correlation between IDSR implementation and meningococcal vaccine importation, we obtained estimates of the doses of vaccine imported by the Burkina Faso government from the WHO International Consultative Group, UNICEF, and GlaxoSmithKline Biologicals. Vaccine data were also collected from the WHO disease outbreak website (http://www.who.int/csr/don/en/index.html) (Tables S3 and S4). We statistically tested if annual importation of doses was impacted by IDSR implementation using the following general linear regression:
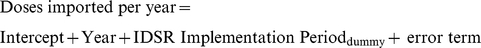
Where IDSR implementation period was recorded as a dummy variable (Pre-IDSR  = 0; Post-IDSR = 1). We ran six models, each time varying the dependent variable (doses imported) as follows: Total annual doses imported (including 1996 data); Total annual doses imported (excluding 1996 data); Doses per 100,000 population in whole country (including 1996 data);

Doses per 100,000 population in whole country (excluding 1996 data); Doses per 100,000 population in districts where outbreaks occurred (including 1996 data); and, doses per 100,000 population in districts where outbreaks occurred (excluding 1996 data). To check for autocorrelation, we calculated the Durbin-Watson statistic for each regression.

### Costs

We used the costs of IDSR-related activities reported in our previous study [26; see also Table S5]. The costs include those due to surveillance and response. In the case of meningitis, response is mostly treatment of those ill and vaccination of populations near a victim (e.g., those in the same village). The cost data for each activity included personnel, transportation items, office consumable goods, public awareness campaigns, laboratory and response materials and supplies, and capital items [26].

We also obtained direct medical care costs incurred by the government to treat a patient with meningitis related-illness at district health facility ($53) and regional hospital ($71) during the 2002 epidemic situation (unpublished data, Ministry of Health, Burkina Faso; see Table S6)). These estimates include only the immediate costs of meningitis case management (e.g., consultation and hospitalization fees, drugs and other essential consumables, and laboratory specimen testing). As the study perspective is the government-funded public health care system, we did not include the costs for transportation to the health facility, provision of long-term care for patients with severe sequelae and loss of productivity due to morbidity and premature death.

### Cost-effectiveness analysis

Assuming that any measured differences between health outcomes (i.e., after IDSR minus before IDSR) are due to IDSR, we calculated the cost-effectiveness in net cost (in dollars) per outcome averted using the following general formula:
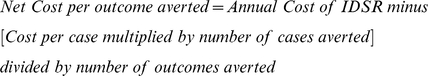
where outcomes averted were cases, deaths or sequelae.

We used the above formula to calculate the cost-effectiveness ratios for the median, 25^th^, and 75^th^ percentiles differences in cases, deaths or sequelae averted before and after IDSR. The median (25^th^, 75^th^) cost effectiveness ratio was calculated using the median (25^th^, 75^th^) annual cost of IDSR activities In this equation, a negative result indicates net cost savings, when the cost savings of IDSR (i.e., cost of treatments avoided due to cases averted) outweigh the costs of IDSR activities (surveillance and response).

We also determined the net cost per capita of IDSR as follow:
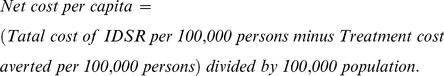



In this equation, the per capita IDSR costs were calculated using data from the whole country [26] while the costs of treatment averted come from districts that reported outbreaks.

### Sensitivity analysis

Regardless of IDSR activities, any measured alteration in the epidemiology of meningitis, including any reduction in cases, could have been due to naturally occurring disease cycles. To remove any unknown effect of disease cycles or specific years, we individually compared health outcomes (incidence of cases, mortality, sequelae, and time-to-peak of outbreak) between each outbreak before IDSR and each outbreak that occurred after IDSR implementation. This analysis removes the element of time sequence, and we only compare outcomes before-versus-after the

start of IDSR. Specifically, we used the following general formula:
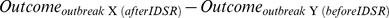
where X is a particular outbreak occurring after IDSR started in 2003, and Y is a particular outbreak before IDSR started.

Using this formula, we generated 9,030 paired comparisons (105 outbreaks before IDSR x 86 outbreaks after IDSR) in outcomes for each of the following variables: incidence of cases, mortality, sequelae, and time-to-peak of outbreak. We also examined the influence of 1996 data on these comparisons by re-running the simulation excluding all outbreaks that occurred in 1996 (giving 7,052 paired comparisons).

We then plotted the distribution of the differences between the paired comparisons (with and without the 1996 data) for the time to peak, and incidence of cases and mortality. We also calculated the median, 25^th^ and 75^th^ percentiles for the cases, deaths and sequelae averted (with and without the 1996 data). Similarly, we calculated the simple average, minimum and maximum of the differences in these health outcomes. Finally, we used the data from the paired comparisons to calculate cost-effectiveness ratios as described earlier.

## Results

### Health outcomes and effectiveness measures

**Figure 1:**
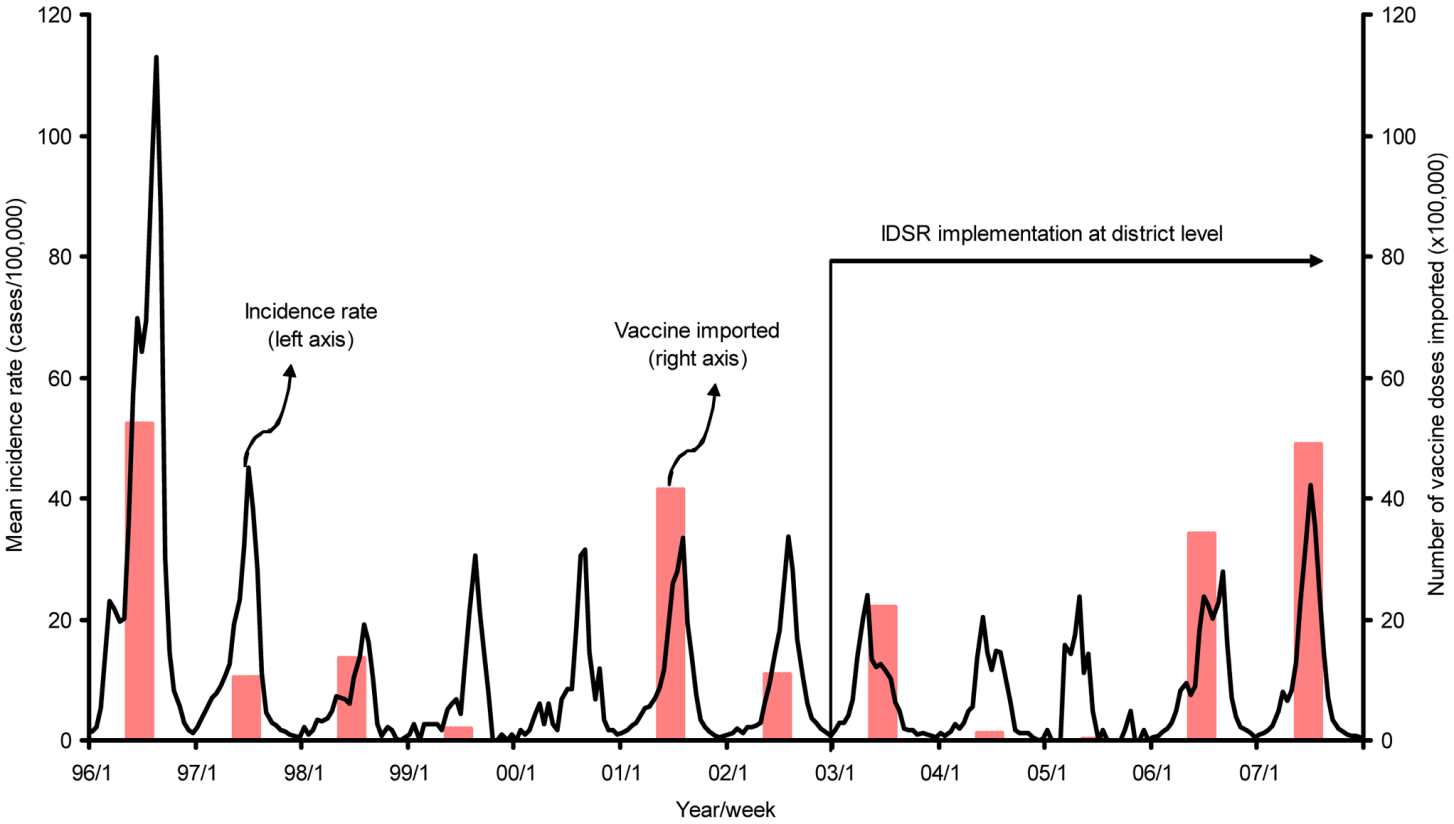
Weekly number of meningococcal meningitis cases (all serotypes) reported during the meningitis season (week 1 to 23) and number of doses of meningococcal vaccine imported from 1996 to 2007 in Burkina Faso. Note: District level weekly new meningitis cases from Burkina Faso for the years 1996–2007 were obtained from the WHO Multi-Diseases Surveillance Center in Ouagadougou, Burkina Faso. Incidence recorded in a particular district experiencing a meningitis outbreak - the incidence data do not apply to the entire country. For list of districts reporting outbreaks recorded in this Figure, see Table S2. Doses of vaccine include all bivalent (AC), trivalent (ACW), and tetravalent (ACWY) polysaccharides imported by the government of Burkina Faso through different organizations (WHO-ICG, UNICEF, MSF, and GlaxoSmithKline Biologicals). Dates of vaccine shipment are representation for purpose of graphing (see also Tables S3 and S4).

Based on our definition of outbreaks (10 per 100,000 – see earlier), we identified 105 outbreaks before adoption of IDSR and 86 after adoption of IDSR (Table S2). The mean weekly meningitis cases per outbreak recorded from 1996 to 2007 at district level in Burkina Faso are represented in Figure 1. Detailed total incidence and mortality, time-to-peak of outbreak, and time to reach a fixed percentage of total incidence and mortality rates per outbreak are showed in Table S2. The size of these outbreaks was variable and 1996 was the largest epidemic year recorded (Figure 1). We observed from Figure 1 some evidence that, after IDSR implementation in 2003, there were lower peaks-of-incidence and possibly lower cumulative total incidence-per-outbreak. However, lower peak incidence after IDSR is not uniform, as we note that the peak incidence in 2007 is greater than the peaks in all but two years (1996 and 1997) before IDSR implementation.

**Figure 2:**
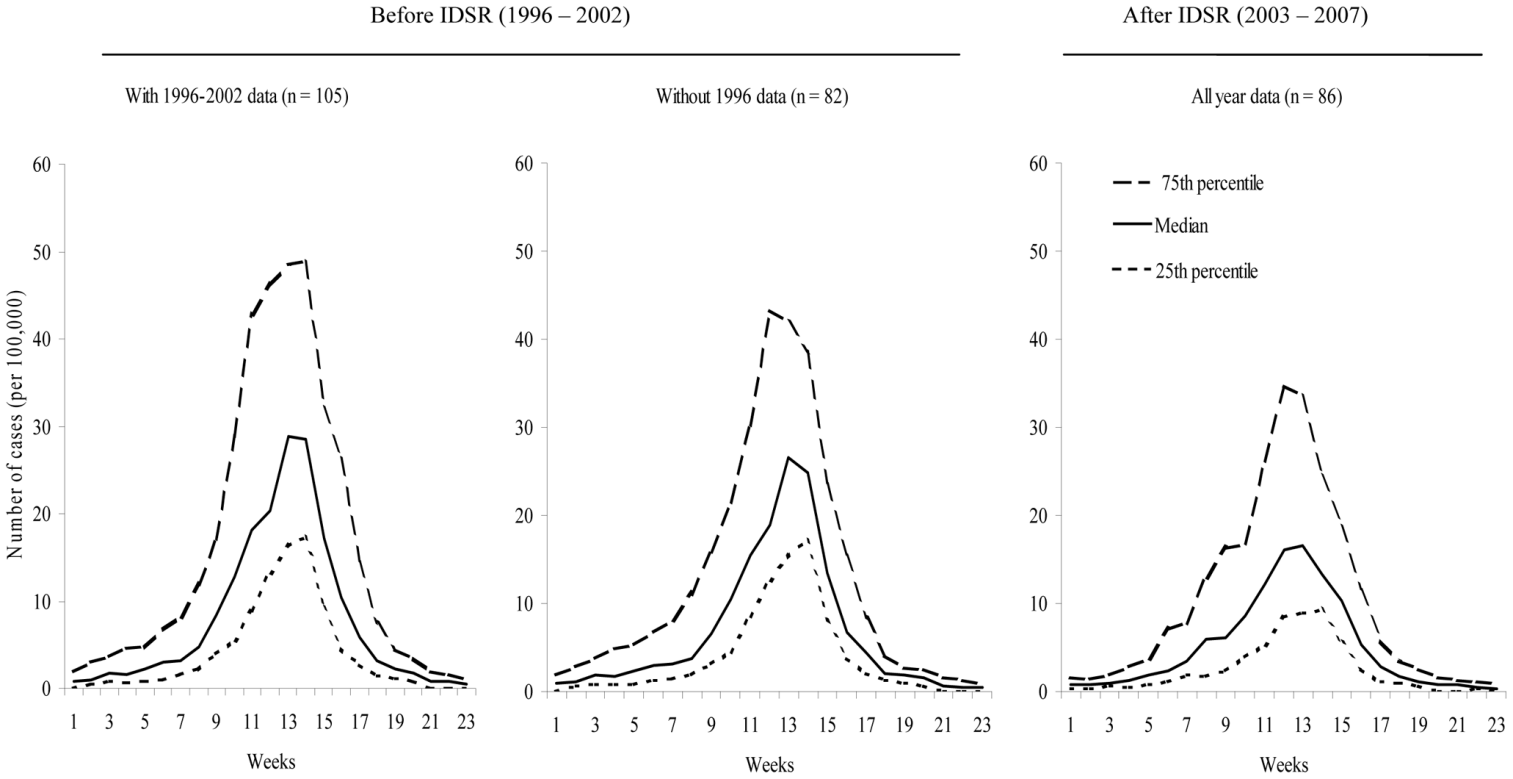
Comparison before (1996–2002) and after (2003–2007) IDSR implementation: weekly median (25^th^ and 75^th^ percentiles) of new cases of meningitis per outbreak. Note: Data source: WHO Multi-Diseases Surveillance Center, Ouagadougou, Burkina Faso. For each weekly incidence rate, we calculated the median, 25^th^ and 75^th^ percentile of the 105 and 82 outbreaks before IDSR and 86 outbreaks after IDSR. Before IDSR, the median (25^th^ and 75^th^ percentile) cumulative number of meningitis cases per outbreak was 185.0 (139.5 and 377.0) per 100,000 inhabitants when 1996 data were included and was 167.9 (135.3 and 281.0) per 100,000 inhabitants when 1996 data were excluded. After IDSR, the median (25^th^ and 75^th^ percentile) cumulative number of deaths per outbreak was 142.0 (100.3 and 248.2) per 100,000 inhabitants.

**Figure 3:**
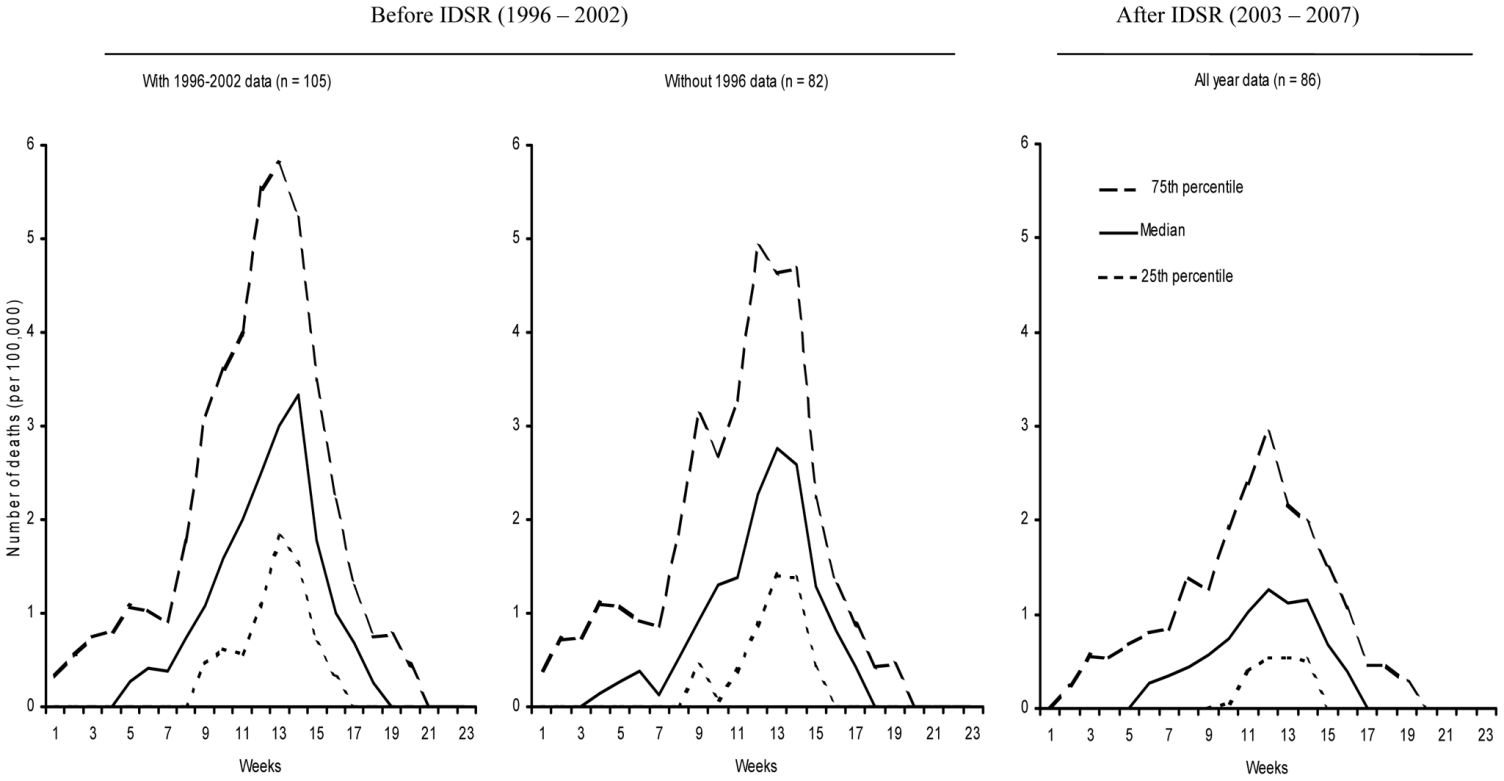
Comparison before (1996–2002) and after (2003–2007) IDSR implementation: weekly median (25^th^ and 75^th^ percentiles) of number of meningitis deaths per outbreak. Data source: WHO Multi-Diseases Surveillance Center, Ouagadougou, Burkina Faso. For each weekly mortality rate, we calculated the median, 25^th^ and 75^th^ percentile of the 105 and 82 outbreaks before IDSR and 86 outbreaks after IDSR. Before IDSR, the median (25^th^ and 75^th^ percentile) cumulative number of deaths per outbreak was 23.8 (17.9 and 35.9) per 100,000 inhabitants when 1996 data were included and was 20.5 (16.1 and 30.3) per 100,000 inhabitants when 1996 data were excluded. After IDSR, the median (25^th^ and 75^th^ percentile) cumulative number of deaths per outbreak was 12.6 (8.8 and 21.3) per 100,000 inhabitants.

The median and the 25^th^ and 75^th^ percentiles of the weekly number of meningitis cases and deaths before and after IDSR implementation are presented in Figures 2 and 3, respectively. The peak of the median value of the number of meningitis cases and deaths after IDSR implementation is lower than the peaks of the before IDSR median values (with and without 1996 data). However, the 75^th^ percentile after IDSR implementation is greater than some of the median values before IDSR, indicating that IDSR implementation does not always equate with lower number of cases (Figures 2 and 3).

The pattern of differences in peaks is also seen when comparing cumulative cases. After IDSR implementation, the average and median (50^th^ percentile) incidences per outbreak dropped by 135 per 100,000 and 40 per 100,000 (p-value 0.0001, Table 1), respectively. Time-to-peak, time to reach a set percentage of total cases, and time to reach a set percentage of total deaths were all reduced after IDSR; however, some p-values of the difference were greater than 0.05 (Table 1). The removal of 1996 data did reduce the size of the differences, but differences did remain, showing that IDSR implementation was correlated with reduction in incidence of cases and mortality, and time-to-peak (Table 1).

**Table 1:** Comparison before (1996–2002) and after (2003–2007) IDSR implementation: Total number of meningitis cases and deaths, time to peak of outbreak, and time to reach total cases and deaths.

		Including 1996 data				Excluding 1996 data			
Outcome measures		Before	After	Difference	p-value*	Before	After	Difference	p-value*
		(n = 105)	(n = 86)			(n = 82)	(n = 86)		
Total cumulative cases (per 100,000)									
Mean		346	211	−135	0.0267	228	211	−17	0.034
25th percentile†		140	100	−40		135	100	−35	
50th percentile†		185	142	−43		168	142	−26	
75th percentile†		377	248	−129		281	248	−33	
Total cumulative deaths (per 100,000)									
Mean		35	16	−19	<0.0001	26	16	−10	<0.0001
25th percentile†		18	9	−9		16	9	−7	
50th percentile†		23	13	−10		21	13	−8	
75th percentile†		36	21	−15		30	21	−9	
Time-to-peak of outbreak§ (weeks)									
Mean		6	4	−2	<0.0001	6	4	−2	<0.0001
25th percentile†		4	3	−1		3	3	0	
50th percentile†		6	4	−2		6	4	−2	
75th percentile†		9	5	−4		8	5	−3	
Time to reach % total cases# (weeks)									
25% of cases¶		10.5	10.2	−0.3	0.1578	10.4	10.2	−0.2	0.2954
50% of cases¶		12.6	12	−0.7	0.0123	12.5	12	−0.5	0.1081
75% of cases¶		14.4	13.6	−0.7	0.015	14.1	13.6	−0.5	0.0404
Time to reach % total deaths# (weeks)									
25% of deaths¶		9.9	9	−0.9	0.0052	9.6	9	−0.6	0.0361
50% of deaths¶		12.2	11.6	−0.6	0.0193	11.9	11.6	−0.3	0.1313
75% of deaths¶		14	13.6	−0.4	0.0609	13.7	13.6	−0.1	0.4307

*p-value of Mann-Whitney score test for two-sample groups. We compared health outcomes for each of the 105 outbreaks before IDSR with each of the 86 outbreaks after IDSR implementation. We then re-ran these paired comparisons excluding the 1996, before IDSR data. Negative figure indicates lower number per outbreak after IDSR than before IDSR implementation.

† These represented the 25^th^ percentile, 50^th^ percentile, and 75^th^ percentile of the total number of cases and deaths and the time to peak before and after IDSR.

§ Time to peak of outbreak represented the time elapsed from reaching the alert threshold of a weekly incidence of 5 cases per 100,000 inhabitants to the week with the maximum weekly incidence.

# Time to reach total cases and deaths represented the time interval between the first week of each calendar year and the week during the outbreak period when the total cases and deaths were reached.

¶ These are the average time for outbreaks to reach the 25th, 50th, and 75^th^ percent of total cases and deaths. For example, before IDSR it took 10.5 weeks during an outbreak to reach 25% of all cases attributed to that outbreak.

Data source: WHO Multi-Disease Surveillance Center, Ouagadougou, Burkina Faso (see Table S2).

### Vaccine imports

We did not find any evidence of a statistically significant correlation associated between doses of vaccine imported and outbreaks (Figure 1, Table S7). There were some obvious increases in recorded importation of vaccine in some of the years that had “large” outbreaks (e.g., 1996, 2001, and 2007). On the other hand, in some years there were little or no recorded imports, regardless of peak incidence (e.g., 1999, 2000, and 2005). We note that we had difficulty in obtaining some of the vaccine import data, particularly for the earlier years examined. We conclude that IDSR has not had an appreciable impact on doses of vaccine imported. Thus, when we considered costs associated with IDSR we did not include any costs associated with increased doses of vaccine used.

### Cost-effectiveness analysis

The median net cost of averting a case of meningitis was $23 per meningitis case averted (25^th^ percentile: $30; 75^th^ percentile: cost savings), and the median cost per death and sequelae averted were $98 and $126, respectively (Table 2). The median cost of operating IDSR was $0.01 per capita (25^th^: $0.01; 75^th^: cost saving) (Table 2). Excluding the 1996 data notably impacted most estimated cost-effectiveness ratios. For example, excluding 1996 data caused the median cost per case averted to approximately quadruple to $80 per case averted (Table 2). We also found that removing the 1996 data increased the median cost per capita to $0.02 (Table 2).

**Table 2:** Cost-effectiveness (2002 US $) correlated with IDSR impact on meningitis cases and deaths averted per outbreak in Burkina Faso.

	Including 1996 data Median*	Excluding 1996 data Median*
	(*25th 75th percentiles*)	(*25^th^ 75th percentiles*)
Total cost of IDSR§	3,684	3,684
Activities (per 100,000)		
Treatment costs#	2,675	1,609
Avoided (per 100,000)	(*2,473 8,018*)	(*2,175 2,037*)
Net IDSR costs	1,009	2,075
(per 100,000)	(*1,211 savings*)	(*1,509 1,647*)
Cost per case	23	80
Averted	(*30 savings*)	(*43 25*)
Cost per death	98	263
Averted	(*140 savings*)	(*207 182*)
Cost per sequelae¶	126	669
Averted	(*212 savings*)	(*296 358*)
Cost per capita	0.01	0.02
	(*0.01 savings*)	(*0.02 0.02*)

Note: We took the perspective of the government-funded public health care system. We compared health outcomes for each of the 105 outbreaks before IDSR with each of the 86 outbreaks after IDSR implementation. We then re-ran these paired comparisons excluding the 1996, before IDSR data.

*The median cost-effectiveness was calculated using the median cost of IDSR activities and the difference in the number of outcomes of the median (25^th^ percentile –75^th^ percentile) outbreak before and the median (25^th^ percentile –75^th^ percentile) after IDSR implementation, respectively.

§ See reference 26. No vaccine cost included because no evidence found of incremental importation of vaccine doses correlated with implementation of IDSR (see Figure 1 and also Table S7).

# We estimated the treatment cost-saving by multiplying the mean medical cost ($62.25) per meningitis patient by the difference in the number of cases per outbreak that occurred before IDSR versus after IDSR.

¶ We calculated the number of sequelae by assuming 20% of all meningitis illness-related survivors have neurological defects.

Data Source: IDSR cost data (see reference 26). We obtained annual population data and district level weekly meningitis cases and deaths from the WhO-MDSC in Ouagadougou, Burkina Faso.

### Sensitivity analysis

**Figure 4:**
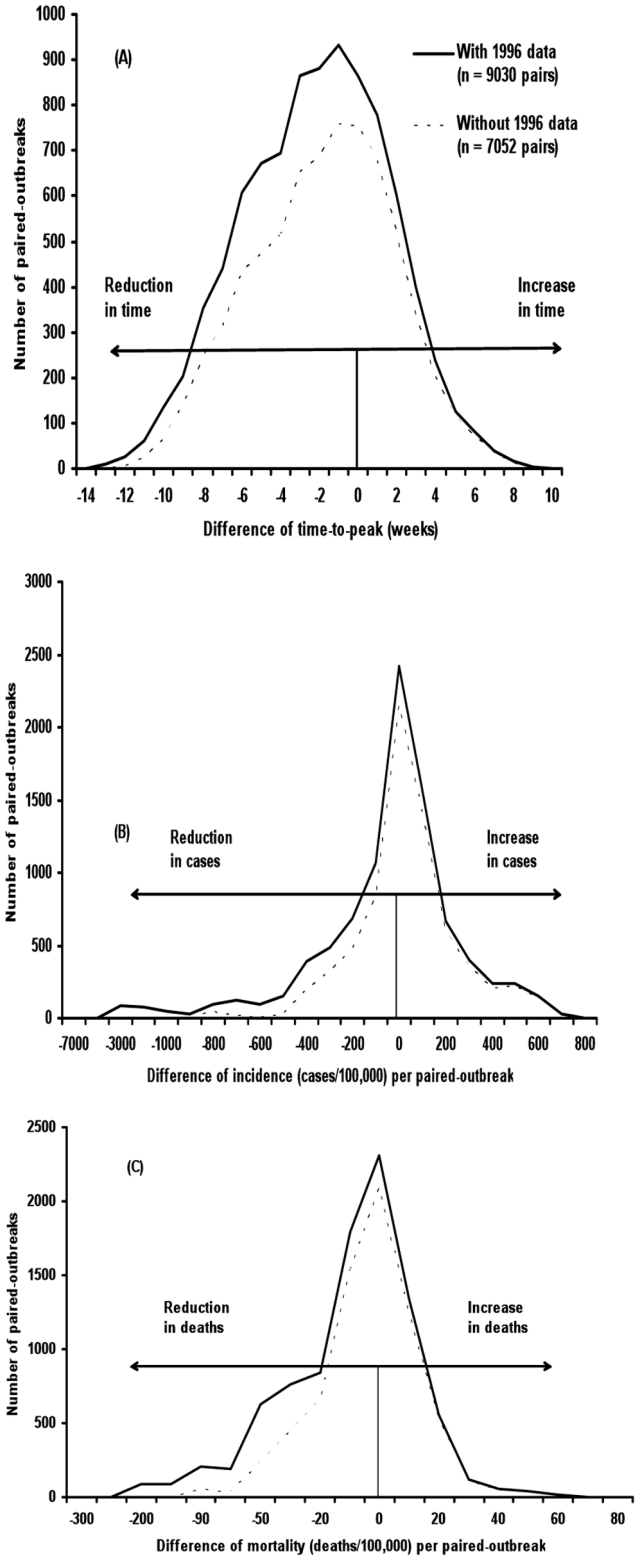
Sensitivity analysis: Distribution of paired comparison of outbreaks before and after IDSR: Difference in time-to-peak, incidence rate, and mortality rate. Note: Comparison of data of each of the 105 outbreaks before IDSR with each of the 86 outbreaks after IDSR. Removing 1996 data reduces total outbreaks before IDSR to 82. Effects on outcomes correlated with IDSR are represented on the horizontal axes: negative numbers indicated reducing effects and positive numbers indicated increasing effects on time-to-peak of outbreak (Panel A), number of cases per outbreak (Panel B), and number of deaths per outbreak (Panel C).

In more than 50% of the pairings in the paired outbreak analysis, which removes the influence of time and disease cycles, the outbreaks after IDSR implementation had lower incidence of cases and mortality, as well as shorter time-to-peak (Figure 4). The median, mean, maximum, and 75^th^ percentiles of all the paired outbreaks all showed a reduction in incidence of cases, deaths and sequelae associated with outbreaks occurring after IDSR implementation (Table 3). However, the data from paired-outbreak comparisons resulted in some lower (i.e., cheaper) median cost-effectiveness estimates (Table 4).

**Table 3:** Sensitivity analysis of number of meningitis cases, deaths and sequelae averted correlated with introduction of IDSR: Paired-comparison of outbreaks before (1996–2002) and after (2003–2007) IDSR implementation:

Outcomes measures#	Including 1996 data†		Excluding 1996 data†	
	Median¶	Average^¶^ *	Median¶	Average^¶^ *
	*(25th percentile 75th percentile)*	*(minimum maximum)*	*(25th percentile 75th percentile)*	*(minimum maximum)*
Total cumulative cases averted (per 100,000)	−48*(58 −201)*	−134*(666 −4,769)*	−27*(80 −115)*	−17*(666 −877)*
Total cumulative deaths averted (per 100,000)	−10*(−1 −25)*	−19*(61 −250)*	−8*(1 −17)*	−10*(61 −102)*
Total cumulative sequelae averted (per 100,000)	−15*(18 −65)*	−44*(267 −2,092)*	−8*(25 −38)*	−6*(267 −375)*

† We compared health outcomes for each of the 105 outbreaks before IDSR with each of the 86 outbreaks after IDSR implementation. We then re-ran these paired comparisons excluding the 1996, before IDSR data.

# Number of health outcomes averted was calculated using the following equation: *Outcome _outbreak X after IDSR_ – Outcome _outbreak Y before IDSR_,* where X for outbreak after IDSR and Y for outbreak before IDSR.

We calculated the number of sequelae by assuming 20% of all meningitis illness-related survivors have neurological defects (see main text).

¶ Negative figure indicates reduction in cases, deaths, and sequelae per outbreak after IDSR implementation.

*These columns present the simple average, minimum and maximum of the differences in health outcomes between paired outbreaks.

Data Source: WHO Multi-Diseases Surveillance Center, Ouagadougou, Burkina Faso.

**Table 4:** Sensitivity analysis of total cost of IDSR, treatment cost of meningitis cases avoided, and cost-effectiveness*: Paired comparison of outbreaks before (1996–2002) and after (2003–2007) IDSR implementation.

	Including 1996 data	Excluding 1996 data
	Median†	Median†
	*(25th percentile 75th percentile)*	*(25th percentile 75th percentile)*
Total cost of IDSR	3,684	3,684
Activities (per 100,000)		
Treatment costs#	2,982	1,662
Avoided (per 100,000)	*(−3,592 12,503)*	(−*4,999 7,159)*
Net IDSR costs	568	2,022
(per 100,000)	*(7,276 savings)*	*(8,683 savings)*
Cost per case	15	76
Averted	*(126 savings)*	*(108 savings)*
Cost per death	68	270
Averted	*(7,276 savings)*	*(6,679 savings)*
Cost per sequelae	46	239
Averted	*(408 savings)*	*(346 savings)*
Cost per capita	0.01	0.02
	*(0.07 savings)*	*(0.09 savings)*

*We took the perspective of the government-funded public health care system. We measured the effectiveness by subtracting the number of cases, deaths, and sequelae of each of the 86 outbreaks after IDSR from each of the 105 outbreaks before IDSR. We then re-ran the analysis excluding the 1996, before IDSR data.

† We calculated the median, 25^th^ and 75^th^ percentile cost-effectiveness based on the difference of the generated health outcomes (after IDSR versus before IDSR) distribution.

# We estimated the treatment costs avoided by multiplying the mean medical cost ($62.25) per meningitis patient by the number of cases averted.

Data Source: WHO Multi-Diseases Surveillance Center, Ouagadougou, Burkina Faso. We calculated the number of sequelae by assuming 20% of all meningitis illness-related survivors have neurological defects.

## Discussion

The IDSR strategy is focused on improving the collection and analysis of infectious disease surveillance data to more rapidly and accurately detect, and respond to, disease outbreaks. This should hopefully reduce the overall burden of disease [14]–[16]. Our results show that, in Burkina Faso, IDSR implementation at a district level was correlated with statistically significant reductions in time-to-peak of outbreak and reductions in total cumulative incidence of cases and mortality. Assuming that such correlations are indicative of causation, our analysis determined that the cost-effectiveness of IDSR ranged from cost savings to cost of $80 per case averted, and from cost savings to $263 per death averted. Our cost-effectiveness estimates were lower (i.e., cheaper), but not dissimilar, than earlier estimates for a meningococcal meningitis mass vaccination campaign in Burkina Faso [21], [30]. These earlier studies estimated US $133 per case and US $2,397 per death averted, uncorrected for inflation [21], [30]. Immunization campaigns in the region for yellow fever, neonatal tetanus, poliomyelitis, and diphtheria reported cost effectiveness ratios of US $281 to $20,591 per death averted [31], [32].

We could not find any statistically significant evidence of an increase in vaccine imports. We can only speculate that the reduced incidence of cases and deaths are due to IDSR allowing public health officials to better target existing resources to outbreaks. That is, the IDSR system enables public health officials to identify and get to an outbreak sooner, and be more certain which villages and areas need interventions.

The study's main limitation is the uncertainty in assuming that the reductions in cases were due to IDSR. We do not have enough evidence to claim the degree that the differences that we measured were due to IDSR. As mentioned earlier, the nature of a public health system such as IDSR prevents us from designing and conducting a controlled scientific experiment. It is possible that the changes in epidemiology that we recorded were due to changes in other factors, such as a change in the circulating serogroups and strains of *Neisseria meningitidis*. For the time period studied, an examination of the WHO data shows that the main circulating strain was serogroup A, although serogroup W135 co-circulated [33, Table S8]. Two studies, which analyzed in greater detail the specimens and data, found that there were some differences in strains (sequence types) of circulating serogroup A over time, with serogroup 7 recorded more frequently after 1995 – though both strains were from the same clonal group [34, 35, Table S8]. However, the sample sizes used in the studies are very small (typically less than 20 isolates per year) and are collected in a non-random fashion (i.e., convenience samples).

Other limitations include the fact that our retrospective survey may not have fully captured all data. This would be due to the limitations of public data records (e.g., no vaccine distribution and use records at country level) or “readily” accessible program records of specific public health projects (such as meningitis specific vaccine program) that run parallel to the national public health system. Further, we assumed that the accuracy of recording cases and deaths due to meningitis remained unchanged during the period studied. The implementation of IDSR could have improved accuracy, potentially increasing the estimated impact of IDSR. However, it is impossible to assess the degree, and therefore impact, of any changes in accuracy over time.

We believe, even with the study limitations, that these results indicate that IDSR is likely to be a cost-effective public health system. A completely accurate accounting of its benefits to society, such as money and lives saved has yet to be documented. It is also clear, however, that IDSR is not a complete solution to eliminating the burden of meningococcal meningitis. Given the difficulty of measuring the impact of surveillance and response systems, it may well be that policy-makers will have to make assessments of the value of IDSR and similar systems using the type of data we presented here.

## Supporting Information

Table S1 List of IDSR priority diseases and diseases of public health importance weekly or monthly reported in Burkina Faso during the study period.
(0.02 MB DOC)

Table S2 Health outcomes and duration of meningococcal meningitis outbreak in selected districts before (1996-2002) and after (2003–2007) IDSR implementation in Burkina Faso.
(0.28 MB DOC)

Table S3 Pattern of annual vaccines imported into Burkina Faso before (1996–2002) and after (2003–2008) IDSR implementation.
(0.04 MB DOC)

Table S4 Pattern of annual vaccines delivered to region before (1996–2002) and after (2003–2008) IDSR implementation.
(0.04 MB DOC)

Table S5 Annual costs (in 2002 US $) of all public health-related surveillance and IDSR-related activities per category of resources and health structure level in Burkina Faso: 2002 to 2005.
(0.07 MB DOC)

Table S6 Cost of treating a meningitis-related illness at regional hospital and district health facility levels in Burkina Faso during the 2002 epidemic season.
(0.03 MB DOC)

Table S7 Results of statistical analyses of doses of vaccine imported into Burkina Faso from 1996 to 2008: Impact of IDSR on amounts of vaccine imported.
(0.03 MB DOC)

Table S8 Pathogens identified by PCR, Latex, and Culture of CSF and serum samples in countries under enhanced surveillance (IDSR) of meningitis in the WHO African region.
(0.17 MB DOC)
