# Supplementary material for: Modeling the Cost-Effectiveness of the Integrated Disease Surveillance and Response (IDSR) System: Meningitis in Burkina Faso
Source: PLoS One. 2010 Sep 28;5(9):e13044. doi: 10.1371/journal.pone.0013044 (PMC2946913; doi:10.1371/journal.pone.0013044)
Supplement: Table S1 — List of IDSR priority diseases and diseases of public health importance weekly or monthly reported in Burkina Faso during the study period. (0.02 MB DOC) [file pone.0013044.s001.doc]

Table S1.

| **IDSR priority diseases and health risk conditions** | | | **Additional diseases selected by national policy** |
| --- | --- | --- | --- |
| **Epidemic-prone diseases** | **Diseases targeted for eradication and elimination** | **Other diseases of public health importance** |
|  |  |  |  |
| Cholera | Poliomyelitis (AFP) | New HIV/AIDS cases | Anthrax |
| Diarrhea with blood (Shigellosis) | Dracunculiasis | Diarrhea with dehydration in < 5-yr old | Diphtheria |
| Measles | Leprosy | Pneumonia in < 5-yr old | Leshmaniasis |
| Meningococcal meningitis | Neonatal tetanus | Malaria | Pertusis |
| Plague |  | Onchocerciasis | Rabies |
| Viral hemorrhagic fevers |  | STIs | Varicella |
| Yellow fever |  | Trypanosomiasis | Viral hepatitis |
|  |  | Tuberculosis |  |

AFP = Acute flaccid paralysis; HIV/AIDS = Human immunodeficiency virus/Acquired Immune Deficiency Syndrome; STIs = Sexually transmitted infections (genital ulcer syndrome and urethral discharge syndrome).

**Source**: Direction de la Lutte contre la Maladie, Ministère de la Santé, Burkina Faso
