# Supplementary material for: Modeling the Cost-Effectiveness of the Integrated Disease Surveillance and Response (IDSR) System: Meningitis in Burkina Faso
Source: PLoS One. 2010 Sep 28;5(9):e13044. doi: 10.1371/journal.pone.0013044 (PMC2946913; doi:10.1371/journal.pone.0013044)
Supplement: Table S2 — Health outcomes and duration of meningococcal meningitis outbreak in selected districts before (1996-2002) and after (2003–2007) IDSR implementation in Burkina Faso. (0.28 MB DOC) [file pone.0013044.s002.doc]

Table S2

|  |  | **Health outcomes** | | | **Duration of outbreak** | | | | | | |
| --- | --- | --- | --- | --- | --- | --- | --- | --- | --- | --- | --- |
| **Year of meningitis outbreak** | **Districts with reported outbreak** | Total cumulative incidence rate (cases per 100,000 inhabitants) | Total cumulative mortality rate (deaths per 100,000 inhabitants) | Total cumulative fatality (%) | Time (weeks) elapsed after the first alert threshold and the peak of outbreak | Time (weeks) elapsed after the first week of the calendar year and the week when % of total cumulative cases was reached | | | Time (weeks) elapsed after the first week of the calendar year and the week when % of total cumulative deaths was reached | | |
|  |  |  |  |  |  |  |  |  |  |  |  |
|  |  |  |  |  |  | 25% | 50% | 75% | 25% | 50% | 75% |
| (Jan - May) |  |  |  |  |  |  |  |  |  |  |  |
| 1996 | Bogande | 732.2 | 85.3 | 11.6 | 8 | 12 | 14 | 16 | 11 | 13 | 15 |
| 1996 | Boulsa | 632.4 | 76.5 | 12.1 | 2 | 11 | 13 | 15 | 11 | 13 | 15 |
| 1996 | Dande | 204.9 | 30.5 | 14.9 | 9 | 11 | 13 | 16 | 10 | 13 | 16 |
| 1996 | Dedougou | 140.1 | 20.3 | 14.5 | 5 | 11 | 13 | 15 | 10 | 12 | 14 |
| 1996 | Diapaga | 968.8 | 104.7 | 10.8 | 7 | 12 | 14 | 15 | 12 | 13 | 15 |
| 1996 | Djibo | 528.7 | 34.5 | 6.5 | 10 | 11 | 14 | 16 | 10 | 13 | 15 |
| 1996 | Dori | 823.6 | 103.8 | 12.6 | 14 | 12 | 15 | 16 | 12 | 15 | 16 |
| 1996 | Fada | 341.0 | 38.2 | 11.2 | 9 | 14 | 15 | 16 | 13 | 15 | 16 |
| 1996 | Kaya | 2545.4 | 182.8 | 7.2 | 11 | 10 | 13 | 15 | 10 | 13 | 15 |
| 1996 | Kombissiri | 864.3 | 109.6 | 12.7 | 12 | 13 | 15 | 16 | 13 | 15 | 16 |
| 1996 | Kongoussi | 1136.9 | 61.9 | 5.4 | 9 | 8 | 10 | 12 | 6 | 10 | 12 |
| 1996 | Koudougou | 477.1 | 64.7 | 13.6 | 3 | 10 | 12 | 14 | 10 | 12 | 14 |
| 1996 | Koupela | 576.2 | 73.3 | 12.7 | 10 | 10 | 13 | 15 | 10 | 13 | 15 |
| 1996 | Leo | 139.5 | 24.9 | 17.9 | 1 | 10 | 12 | 14 | 10 | 12 | 15 |
| 1996 | Manga | 98.0 | 13.2 | 13.4 | 5 | 11 | 14 | 16 | 13 | 14 | 16 |
| 1996 | Nouna | 185.0 | 21.1 | 11.4 | 10 | 10 | 14 | 16 | 9 | 13 | 16 |
| 1996 | Ouahigouya | 4832.4 | 253.7 | 5.2 | 14 | 7 | 12 | 15 | 8 | 11 | 15 |
| 1996 | Reo | 337.4 | 53.0 | 15.7 | 6 | 12 | 14 | 15 | 11 | 14 | 15 |
| 1996 | Tenkodogo | 375.4 | 59.0 | 15.7 | 6 | 11 | 14 | 15 | 11 | 13 | 15 |
| 1996 | Tougan | 279.1 | 32.0 | 11.5 | 5 | 13 | 15 | 16 | 12 | 14 | 15 |
| 1996 | Yako | 256.4 | 22.0 | 8.6 | 7 | 12 | 14 | 15 | 10 | 13 | 15 |
| 1996 | Ziniare | 498.7 | 69.0 | 13.8 | 6 | 12 | 14 | 16 | 12 | 13 | 15 |
| 1996 | Zorgho | 600.7 | 63.4 | 10.6 | 7 | 12 | 15 | 16 | 12 | 15 | 16 |
| 1997 | Banfora | 122.4 | 9.2 | 7.5 | 3 | 12 | 13 | 14 | 10 | 12 | 14 |
| 1997 | Barsalgo | 116.4 | 10.3 | 8.9 | 5 | 10 | 12 | 13 | 10 | 11 | 13 |
| 1997 | Bogande | 151.1 | 21.5 | 14.3 | 2 | 4 | 6 | 12 | 4 | 7 | 12 |
| 1997 | Boromo | 482.8 | 62.4 | 12.9 | 8 | 10 | 12 | 13 | 10 | 12 | 13 |
| 1997 | Dande | 300.0 | 19.2 | 6.4 | 6 | 11 | 12 | 13 | 11 | 12 | 13 |
| 1997 | Dano | 318.1 | 29.9 | 9.4 | 7 | 10 | 11 | 13 | 9 | 10 | 12 |
| 1997 | Dedougou | 167.4 | 21.5 | 12.9 | 2 | 11 | 12 | 14 | 10 | 12 | 14 |
| 1997 | Diapaga | 162.1 | 20.8 | 12.8 | 2 | 4 | 7 | 11 | 4 | 6 | 11 |
| 1997 | Fada | 365.2 | 47.4 | 13.0 | 9 | 6 | 9 | 12 | 5 | 9 | 12 |
| 1997 | Hounde | 483.8 | 27.7 | 5.7 | 10 | 8 | 11 | 13 | 8 | 11 | 13 |
| 1997 | Kombissiri | 136.9 | 15.5 | 11.3 | 5 | 7 | 9 | 11 | 8 | 9 | 10 |
| 1997 | Kossodo | 154.3 | 13.6 | 8.8 | 4 | 3 | 11 | 14 | 11 | 13 | 16 |
| 1997 | Koudougou | 220.8 | 32.4 | 14.7 | 7 | 11 | 12 | 14 | 9 | 12 | 14 |
| 1997 | Koupela | 279.8 | 37.7 | 13.5 | 10 | 7 | 11 | 12 | 7 | 11 | 13 |
| 1997 | Leo | 128.2 | 26.2 | 20.4 | 12 | 10 | 12 | 14 | 9 | 11 | 13 |
| 1997 | Manga | 583.4 | 60.5 | 10.4 | 10 | 9 | 11 | 13 | 8 | 10 | 12 |
| 1997 | Nouna | 248.6 | 30.9 | 12.4 | 13 | 11 | 13 | 14 | 9 | 12 | 14 |
| 1997 | Orodara | 255.7 | 28.5 | 11.1 | 2 | 12 | 13 | 14 | 11 | 12 | 14 |
| 1997 | Ouargaye | 468.5 | 46.3 | 9.9 | 11 | 6 | 12 | 12 | 8 | 11 | 12 |
| 1997 | Pama | 219.9 | 15.8 | 7.2 | 9 | 8 | 11 | 13 | 5 | 10 | 11 |
| 1997 | Paul VI | 135.2 | 16.0 | 11.9 | 4 | 11 | 13 | 15 | 12 | 13 | 14 |
| 1997 | Pissy | 92.5 | 8.9 | 9.6 | 3 | 12 | 13 | 14 | 12 | 13 | 14 |
| 1997 | Po | 559.0 | 65.0 | 11.6 | 10 | 7 | 11 | 13 | 6 | 10 | 12 |
| 1997 | Reo | 940.3 | 105.6 | 11.2 | 7 | 9 | 11 | 13 | 8 | 10 | 12 |
| 1997 | Secteur15 | 132.0 | 10.7 | 8.1 | 3 | 12 | 13 | 13 | 12 | 12 | 12 |
| 1997 | Secteur22 | 219.2 | 2.9 | 1.3 | 3 | 12 | 13 | 14 | 12 | 13 | 13 |
| 1997 | Sindou | 155.0 | 21.4 | 13.8 | 3 | 13 | 14 | 17 | 12 | 13 | 15 |
| 1997 | Solenzo | 138.4 | 17.9 | 12.9 | 4 | 11 | 13 | 14 | 12 | 13 | 14 |
| 1997 | Tenkodogo | 584.1 | 76.0 | 13.0 | 12 | 8 | 10 | 12 | 7 | 10 | 12 |
| 1997 | Toma | 281.4 | 25.9 | 9.2 | 10 | 8 | 11 | 13 | 9 | 11 | 12 |
| 1997 | Tougan | 253.0 | 19.0 | 7.5 | 9 | 10 | 12 | 13 | 11 | 12 | 13 |
| 1997 | Yako | 135.6 | 10.2 | 7.6 | 3 | 12 | 13 | 14 | 12 | 13 | 14 |
| 1997 | Zabre | 227.5 | 29.6 | 13.0 | 4 | 10 | 11 | 13 | 10 | 11 | 13 |
| 1997 | Ziniare | 89.3 | 12.2 | 13.7 | 5 | 10 | 12 | 14 | 9 | 11 | 14 |
| 1997 | Zorgho | 167.2 | 21.1 | 12.6 | 7 | 9 | 11 | 12 | 7 | 10 | 12 |
| 1998 | Boromo | 90.4 | 9.2 | 10.2 | 8 | 8 | 13 | 15 | 7 | 14 | 15 |
| 1998 | Sindou | 160.3 | 25.7 | 16.0 | 7 | 10 | 13 | 15 | 9 | 14 | 15 |
| 1999 | Sebba | 146.8 | 22.9 | 15.6 | 6 | 12 | 15 | 16 | 10 | 14 | 16 |
| 2000 | Sebba | 172.3 | 23.8 | 13.8 | 10 | 13 | 15 | 16 | 13 | 15 | 16 |
| 2001 | Barsalgo | 205.1 | 31.0 | 15.1 | 2 | 13 | 14 | 16 | 10 | 13 | 14 |
| 2001 | Bogande | 403.4 | 58.2 | 14.4 | 12 | 8 | 11 | 13 | 6 | 10 | 13 |
| 2001 | Boromo | 178.3 | 29.8 | 16.7 | 2 | 12 | 13 | 14 | 12 | 13 | 14 |
| 2001 | Boulsa | 301.7 | 34.2 | 11.3 | 7 | 10 | 12 | 14 | 9 | 11 | 12 |
| 2001 | Dano | 129.8 | 35.9 | 27.7 | 11 | 4 | 9 | 12 | 3 | 7 | 12 |
| 2001 | Diapaga | 390.4 | 25.2 | 6.5 | 8 | 11 | 12 | 14 | 10 | 12 | 14 |
| 2001 | Dori | 158.3 | 19.0 | 12.0 | 9 | 13 | 15 | 16 | 12 | 15 | 16 |
| 2001 | Fada | 162.1 | 16.6 | 10.2 | 5 | 10 | 12 | 14 | 10 | 11 | 13 |
| 2001 | Gaoua | 143.1 | 22.0 | 15.4 | 4 | 6 | 8 | 10 | 6 | 8 | 10 |
| 2001 | Gorom | 179.3 | 18.4 | 10.2 | 4 | 11 | 13 | 16 | 13 | 14 | 16 |
| 2001 | Kaya | 134.2 | 20.2 | 15.0 | 3 | 13 | 14 | 15 | 13 | 14 | 15 |
| 2001 | Kossodo | 183.9 | 11.1 | 6.0 | 7 | 12 | 14 | 15 | 7 | 10 | 14 |
| 2001 | Koudougou | 77.0 | 17.4 | 22.6 | 0 | 10 | 14 | 16 | 7 | 14 | 16 |
| 2001 | Koupela | 427.3 | 54.0 | 12.6 | 9 | 10 | 12 | 14 | 9 | 12 | 13 |
| 2001 | Ouargaye | 131.0 | 17.5 | 13.4 | 8 | 10 | 12 | 14 | 7 | 12 | 14 |
| 2001 | Pama | 168.4 | 19.8 | 11.8 | 6 | 11 | 13 | 15 | 12 | 12 | 14 |
| 2001 | Paul VI | 166.0 | 8.2 | 4.9 | 3 | 11 | 12 | 14 | 11 | 13 | 14 |
| 2001 | Pissy | 160.9 | 16.3 | 10.2 | 5 | 12 | 14 | 16 | 12 | 14 | 16 |
| 2001 | Sebba | 181.6 | 30.5 | 16.8 | 6 | 9 | 12 | 13 | 8 | 10 | 13 |
| 2001 | Secteur30 | 377.7 | 30.5 | 8.1 | 10 | 12 | 14 | 16 | 11 | 15 | 16 |
| 2001 | Sindou | 83.4 | 17.1 | 20.5 | 3 | 11 | 12 | 14 | 9 | 12 | 14 |
| 2001 | Tenkodogo | 206.1 | 18.9 | 9.2 | 6 | 12 | 13 | 14 | 11 | 13 | 14 |
| 2001 | Ziniare | 54.9 | 11.3 | 20.6 | 1 | 13 | 14 | 16 | 13 | 14 | 15 |
| 2001 | Zorgho | 377.0 | 60.3 | 16.0 | 11 | 11 | 13 | 14 | 11 | 12 | 14 |
| 2002 | Bousse | 281.8 | 26.9 | 9.6 | 7 | 12 | 14 | 15 | 13 | 14 | 15 |
| 2002 | Diebougou | 111.7 | 18.6 | 16.7 | 6 | 8 | 10 | 14 | 6 | 9 | 12 |
| 2002 | Fada | 99.8 | 14.0 | 14.0 | 7 | 12 | 14 | 15 | 10 | 14 | 16 |
| 2002 | Kaya | 144.1 | 19.4 | 13.4 | 5 | 11 | 13 | 15 | 10 | 12 | 14 |
| 2002 | Kossodo | 102.0 | 9.8 | 9.6 | 2 | 11 | 13 | 15 | 9 | 13 | 14 |
| 2002 | Koudougou | 108.1 | 15.4 | 14.2 | 3 | 11 | 14 | 15 | 6 | 13 | 14 |
| 2002 | Koupela | 119.6 | 18.9 | 15.8 | 4 | 10 | 12 | 14 | 10 | 11 | 14 |
| 2002 | Manga | 86.8 | 9.8 | 11.2 | 1 | 13 | 14 | 16 | 13 | 13 | 15 |
| 2002 | Nanoro | 313.5 | 33.8 | 10.8 | 3 | 12 | 13 | 15 | 12 | 13 | 14 |
| 2002 | Pama | 156.3 | 26.4 | 16.9 | 6 | 10 | 11 | 14 | 11 | 12 | 13 |
| 2002 | Paul VI | 250.9 | 20.2 | 8.0 | 4 | 14 | 16 | 17 | 14 | 14 | 17 |
| 2002 | Pissy | 423.8 | 19.8 | 4.7 | 6 | 12 | 14 | 15 | 10 | 13 | 15 |
| 2002 | Reo | 180.3 | 22.8 | 12.6 | 4 | 12 | 14 | 15 | 11 | 13 | 15 |
| 2002 | Sapone | 214.6 | 34.6 | 16.1 | 9 | 13 | 14 | 15 | 13 | 14 | 15 |
| 2002 | Secteur30 | 174.5 | 15.8 | 9.0 | 4 | 13 | 14 | 16 | 11 | 14 | 14 |
| 2002 | Toma | 71.1 | 13.4 | 18.8 | 1 | 13 | 14 | 16 | 13 | 14 | 15 |
| 2002 | Yako | 555.5 | 34.9 | 6.3 | 5 | 12 | 14 | 15 | 10 | 12 | 14 |
| 2002 | Ziniare | 151.4 | 18.1 | 12.0 | 5 | 11 | 13 | 15 | 10 | 13 | 15 |
| 2002 | Zorgho | 136.2 | 18.3 | 13.5 | 5 | 11 | 13 | 14 | 9 | 12 | 14 |
| Start of IDSR |  |  |  |  |  |  |  |  |  |  |  |
| 2003 | Batie | 383.6 | 33.4 | 8.7 | 4 | 7 | 8 | 9 | 7 | 8 | 11 |
| 2003 | Bogande | 112.8 | 11.0 | 9.8 | 4 | 7 | 9 | 11 | 6 | 8 | 10 |
| 2003 | Boulsa | 77.9 | 10.6 | 13.6 | 5 | 8 | 11 | 13 | 9 | 10 | 13 |
| 2003 | Leo | 107.9 | 23.3 | 21.6 | 4 | 5 | 6 | 10 | 5 | 6 | 10 |
| 2003 | Manga | 218.9 | 13.9 | 6.3 | 4 | 7 | 8 | 10 | 6 | 8 | 10 |
| 2003 | Pama | 67.4 | 11.9 | 17.6 | 2 | 7 | 8 | 12 | 7 | 10 | 14 |
| 2003 | Po | 173.2 | 24.7 | 14.3 | 7 | 8 | 11 | 13 | 8 | 10 | 13 |
| 2003 | Sapone | 96.0 | 11.2 | 11.7 | 4 | 5 | 7 | 11 | 6 | 6 | 10 |
| 2003 | Tenkodogo | 101.5 | 8.8 | 8.7 | 9 | 8 | 12 | 14 | 7 | 12 | 14 |
| 2003 | Zabre | 225.5 | 26.3 | 11.7 | 5 | 10 | 11 | 12 | 7 | 11 | 14 |
| 2004 | Diebougou | 330.0 | 27.7 | 8.4 | 4 | 9 | 10 | 13 | 9 | 10 | 13 |
| 2004 | Gaoua | 77.9 | 8.2 | 10.5 | 1 | 11 | 13 | 14 | 6 | 12 | 15 |
| 2004 | Nanoro | 116.4 | 27.8 | 23.9 | 7 | 9 | 12 | 14 | 8 | 12 | 14 |
| 2004 | Toma | 74.1 | 10.2 | 13.8 | 2 | 13 | 15 | 16 | 7 | 14 | 15 |
| 2004 | Zabre | 70.6 | 19.0 | 26.9 | 4 | 8 | 11 | 13 | 6 | 10 | 13 |
| 2005 | Batie | 112.5 | 6.3 | 5.6 | 3 | 6 | 8 | 9 | 6 | 7 | 8 |
| 2006 | Banfora | 112.8 | 20.6 | 18.2 | 3 | 7 | 9 | 11 | 7 | 9 | 11 |
| 2006 | Barsalogo | 342.3 | 27.1 | 7.9 | 4 | 11 | 13 | 16 | 11 | 14 | 15 |
| 2006 | Boromo | 70.7 | 10.2 | 14.5 | 4 | 9 | 11 | 13 | 5 | 11 | 12 |
| 2006 | Boulsa | 88.1 | 9.4 | 10.6 | 5 | 12 | 15 | 17 | 12 | 16 | 17 |
| 2006 | Bousse | 721.3 | 63.4 | 8.8 | 6 | 13 | 15 | 16 | 14 | 14 | 16 |
| 2006 | Dande | 116.9 | 11.8 | 10.1 | 2 | 12 | 13 | 14 | 11 | 13 | 15 |
| 2006 | Dano | 234.3 | 22.5 | 9.6 | 4 | 7 | 8 | 11 | 7 | 8 | 11 |
| 2006 | Dedougou | 106.4 | 12.4 | 11.7 | 4 | 12 | 14 | 15 | 10 | 14 | 15 |
| 2006 | Diebougou | 134.7 | 15.4 | 11.5 | 6 | 11 | 12 | 13 | 8 | 12 | 12 |
| 2006 | Djibo | 125.8 | 11.5 | 9.2 | 3 | 12 | 13 | 14 | 12 | 14 | 16 |
| 2006 | Fada | 99.6 | 5.7 | 5.7 | 4 | 13 | 16 | 17 | 14 | 15 | 16 |
| 2006 | Gaoua | 193.8 | 16.0 | 8.3 | 9 | 7 | 10 | 12 | 7 | 9 | 12 |
| 2006 | Gourcy | 169.6 | 18.0 | 10.6 | 3 | 7 | 9 | 13 | 7 | 8 | 14 |
| 2006 | Hounde | 250.2 | 20.1 | 8.0 | 4 | 10 | 12 | 13 | 11 | 12 | 12 |
| 2006 | Kaya | 221.0 | 14.7 | 6.6 | 5 | 11 | 12 | 15 | 11 | 12 | 14 |
| 2006 | Kongoussi | 424.2 | 31.0 | 7.3 | 5 | 8 | 10 | 14 | 8 | 11 | 13 |
| 2006 | Koudougou | 181.4 | 13.9 | 7.7 | 3 | 13 | 14 | 15 | 11 | 14 | 15 |
| 2006 | Leo | 79.7 | 13.2 | 16.6 | 2 | 12 | 14 | 15 | 8 | 13 | 15 |
| 2006 | Nanoro | 393.3 | 26.6 | 6.8 | 4 | 15 | 16 | 17 | 15 | 16 | 17 |
| 2006 | Nouna | 133.4 | 7.8 | 5.8 | 4 | 14 | 15 | 16 | 11 | 14 | 15 |
| 2006 | Ouahigouya | 90.9 | 7.0 | 7.7 | 4 | 10 | 11 | 13 | 9 | 11 | 13 |
| 2006 | Pama | 78.2 | 6.0 | 7.7 | 2 | 12 | 13 | 15 | 3 | 10 | 12 |
| 2006 | Paul VI | 210.8 | 12.2 | 5.8 | 4 | 15 | 16 | 18 | 11 | 16 | 16 |
| 2006 | Sapouy | 75.5 | 15.0 | 19.8 | 2 | 12 | 14 | 15 | 12 | 14 | 15 |
| 2006 | Secteur15 | 558.2 | 22.9 | 4.1 | 9 | 9 | 12 | 13 | 9 | 12 | 13 |
| 2006 | Seguenega | 338.1 | 28.9 | 8.5 | 4 | 11 | 12 | 15 | 9 | 12 | 15 |
| 2006 | Sindou | 85.2 | 8.8 | 10.3 | 5 | 8 | 11 | 12 | 5 | 10 | 12 |
| 2006 | Solenzo | 242.4 | 19.9 | 8.2 | 9 | 8 | 11 | 12 | 8 | 11 | 12 |
| 2006 | Titao | 571.4 | 21.4 | 3.7 | 6 | 16 | 17 | 18 | 12 | 16 | 18 |
| 2006 | Toma | 63.0 | 6.6 | 10.5 | 4 | 12 | 15 | 16 | 4 | 14 | 17 |
| 2006 | Tougan | 108.9 | 11.0 | 10.1 | 2 | 13 | 14 | 16 | 12 | 14 | 15 |
| 2006 | Yako | 273.6 | 21.8 | 8.0 | 6 | 14 | 15 | 16 | 14 | 15 | 16 |
| 2006 | Ziniare | 170.1 | 10.7 | 6.3 | 2 | 12 | 13 | 13 | 11 | 13 | 13 |
| 2007 | Banfora | 148.9 | 12.6 | 8.5 | 7 | 7 | 10 | 12 | 7 | 11 | 14 |
| 2007 | Barsalgo | 138.0 | 4.0 | 2.9 | 4 | 11 | 13 | 15 | 10 | 10 | 13 |
| 2007 | Batie | 99.9 | 6.1 | 6.1 | 3 | 5 | 7 | 10 | 3 | 5 | 9 |
| 2007 | Bogande | 168.7 | 7.5 | 4.4 | 4 | 12 | 13 | 14 | 11 | 13 | 15 |
| 2007 | Boulsa | 139.5 | 7.6 | 5.5 | 3 | 8 | 10 | 13 | 9 | 11 | 14 |
| 2007 | Bousse | 301.8 | 27.6 | 9.2 | 6 | 10 | 12 | 14 | 9 | 12 | 14 |
| 2007 | Dande | 90.1 | 9.3 | 10.3 | 3 | 11 | 13 | 15 | 8 | 11 | 14 |
| 2007 | Dano | 144.6 | 18.5 | 12.8 | 6 | 9 | 11 | 13 | 9 | 11 | 13 |
| 2007 | Dori | 102.8 | 6.8 | 6.6 | 3 | 13 | 15 | 16 | 11 | 14 | 17 |
| 2007 | Fada | 233.2 | 8.5 | 3.7 | 4 | 9 | 10 | 12 | 9 | 10 | 13 |
| 2007 | Gaoua | 97.3 | 6.7 | 6.9 | 5 | 8 | 10 | 12 | 7 | 9 | 14 |
| 2007 | Gorom | 125.6 | 12.8 | 10.2 | 5 | 13 | 15 | 16 | 12 | 15 | 16 |
| 2007 | Kaya | 98.9 | 4.5 | 4.6 | 3 | 11 | 14 | 15 | 9 | 14 | 16 |
| 2007 | Kombissiri | 690.9 | 26.4 | 3.8 | 3 | 10 | 11 | 11 | 10 | 11 | 12 |
| 2007 | Kongoussi | 131.4 | 7.0 | 5.4 | 4 | 10 | 12 | 14 | 7 | 11 | 14 |
| 2007 | Kossodo | 123.3 | 6.5 | 5.3 | 2 | 11 | 12 | 14 | 11 | 12 | 14 |
| 2007 | Koudougou | 75.0 | 6.9 | 9.2 | 2 | 11 | 13 | 14 | 6 | 11 | 13 |
| 2007 | Koupela | 263.8 | 16.1 | 6.1 | 4 | 11 | 12 | 13 | 10 | 11 | 13 |
| 2007 | Leo | 227.7 | 21.0 | 9.2 | 7 | 11 | 13 | 14 | 11 | 13 | 14 |
| 2007 | Manga | 115.3 | 10.2 | 8.9 | 2 | 12 | 13 | 14 | 12 | 13 | 14 |
| 2007 | Nanoro | 679.9 | 48.7 | 7.2 | 8 | 11 | 13 | 14 | 10 | 12 | 13 |
| 2007 | Orodara | 162.1 | 7.9 | 4.9 | 1 | 12 | 13 | 14 | 12 | 12 | 14 |
| 2007 | Ouargaye | 534.7 | 33.8 | 6.3 | 5 | 5 | 6 | 9 | 5 | 6 | 8 |
| 2007 | Pama | 434.0 | 10.0 | 2.3 | 4 | 12 | 12 | 13 | 11 | 12 | 13 |
| 2007 | Paul VI | 219.7 | 12.6 | 5.7 | 3 | 11 | 12 | 14 | 11 | 13 | 15 |
| 2007 | Pissy | 351.5 | 19.3 | 5.5 | 3 | 11 | 12 | 13 | 11 | 12 | 13 |
| 2007 | Po | 65.8 | 9.3 | 14.2 | 1 | 10 | 13 | 14 | 7 | 9 | 14 |
| 2007 | Reo | 264.0 | 17.2 | 6.5 | 4 | 11 | 13 | 14 | 9 | 11 | 13 |
| 2007 | Sapone | 597.8 | 36.1 | 6.0 | 3 | 10 | 11 | 12 | 11 | 11 | 12 |
| 2007 | Secteur30 | 576.8 | 30.4 | 5.3 | 4 | 11 | 12 | 13 | 10 | 12 | 12 |
| 2007 | Seguenega | 121.3 | 8.9 | 7.3 | 4 | 12 | 14 | 15 | 10 | 14 | 15 |
| 2007 | Tenkodogo | 210.0 | 14.9 | 7.1 | 5 | 10 | 12 | 13 | 11 | 12 | 13 |
| 2007 | Titao | 129.1 | 12.3 | 9.5 | 1 | 6 | 10 | 14 | 6 | 11 | 14 |
| 2007 | Toma | 182.2 | 14.0 | 7.7 | 3 | 12 | 13 | 14 | 6 | 13 | 15 |
| 2007 | Yako | 451.6 | 28.4 | 6.3 | 6 | 11 | 12 | 14 | 10 | 12 | 14 |
| 2007 | Ziniare | 79.8 | 6.7 | 8.5 | 1 | 12 | 13 | 14 | 12 | 12 | 15 |
| 2007 | Zorgho | 171.3 | 11.5 | 6.7 | 6 | 10 | 13 | 14 | 9 | 12 | 13 |
|  |  |  |  |  |  |  |  |  |  |  |  |
| **Before IDSR** | Mean | 345.5 | 35.4 | 12.1 | 6 | 10 | 13 | 14 | 9.9 | 12.2 | 14.0 |
| (1996 - 2002) | 25th percentile | 139.5 | 17.5 | 9.6 | 4 | 10 | 12 | 14 | 9 | 11 | 13 |
|  | 50th percentile | 185.0 | 22.9 | 12.1 | 6 | 11 | 13 | 14 | 10 | 13 | 14 |
|  | 75th percentile | 377.0 | 35.9 | 14.2 | 9 | 12 | 14 | 15 | 12 | 13 | 15 |
|  |  |  |  |  |  |  |  |  |  |  |  |
| **After IDSR** | Mean | 211.2 | 16.1 | 9.0 | 4 | 10 | 12 | 14 | 9.0 | 11.6 | 13.6 |
| (2003 - 2007) | 25th percentile | 100.3 | 8.8 | 6.1 | 3 | 8 | 11 | 13 | 7 | 10 | 13 |
|  | 50th percentile | 142.0 | 12.6 | 8.1 | 4 | 11 | 12 | 14 | 9 | 12 | 14 |
|  | 75th percentile | 248.2 | 21.3 | 10.3 | 5 | 12 | 13 | 15 | 11 | 13 | 15 |
