# Supplementary material for: Modeling the Cost-Effectiveness of the Integrated Disease Surveillance and Response (IDSR) System: Meningitis in Burkina Faso
Source: PLoS One. 2010 Sep 28;5(9):e13044. doi: 10.1371/journal.pone.0013044 (PMC2946913; doi:10.1371/journal.pone.0013044)
Supplement: Table S3 — Pattern of annual vaccines imported into Burkina Faso before (1996–2002) and after (2003–2008) IDSR implementation. (0.04 MB DOC) [file pone.0013044.s003.doc]

Table S3:

| Time Period | | **Sources of vaccine imported into Burkina Faso** | | | | | | |
| --- | --- | --- | --- | --- | --- | --- | --- | --- |
|  |  | **WHO ICG*** | **As reported on:**  **WHO website**** | **GlaxoSmithKline Biologicals** | **UNICEF*** | **TOTAL** | **Doses per 100,000 inhabitants** | |
| **Before or after IDSR** | **Year** |  |  |  |  |  | **Total country** | **Only in districts with outbreaks** |
| Before IDSR implementation at district level | 1996 |  | 70,000 | 5,229,750 |  | 5,299,750 | 50,136 | 82,303 |
|  | 1997 |  |  | 1,051,500 |  | 1,051,500 | 9,668 | 12,852 |
|  | 1998 | 0 |  | 1,350,000 |  | 1,350,000 | 12,062 | 152,159 |
|  | 1999 | 0 |  | 10,000 | 185,000 | 195,000 | 1,692 | 57,591 |
|  | 2000 | 0 |  | 0 |  | 0 | 0 | 0 |
|  | 2001 | 2,374,000 | 1,760,000 | 0 |  | 4,134,000 | 33,714 | 72,915 |
|  | 2002 | 25,000 | 946,000 | 111,320 |  | 1,082,320 | 8,546 | 20,142 |
|  |  |  |  |  |  |  |  |  |
| IDSR implementation at district level | 2003 | 2,000,000 |  | 217,500 |  | 2,217,500 | 16,951 | 116,584 |
|  | 2004 | 130,000 |  | 260 |  | 130,260 | 964 | 17,093 |
|  | 2005 | 0 |  | 32,060 |  | 32,060 | 230 | 50,799 |
|  | 2006 | 3,383,305 |  | 37,000 |  | 3,420,305 | 23,821 | 43,202 |
|  | 2007 | 3,826,665 |  | 44,240 | 1,031,300 | 4,902,205 | 33,174 | 49,783 |
|  | 2008 | 563,220 |  | 10,930 |  | 574,150 | 3,901 |  |

The mean (median) number of imported doses per 100,000 inhabitants in the country and only districts in outbreaks was 16,546 (9,668) and 56,852 (57,591) before IDSR versus 13,173 (10,426) and 55,492 (49,783) after IDSR implementation, respectively.

*Acronyms: WHO – World Health Organization; ICG - International Consultative Group; UNICEF – United Nations Children’s Fund.

** WHO website <http://www.who.int/csr/don/archive/country/bfa/en/> - accessed September 05, 2010.

**Source:** Doses of vaccine delivered to the government of Burkina Faso by the WHO International Consultative Group, UNICEF, and GlaxoSmithKline Biologicals. Supplementary vaccine data for intervention were collected from the WHO disease outbreak website (see above)
