# Supplementary material for: Modeling the Cost-Effectiveness of the Integrated Disease Surveillance and Response (IDSR) System: Meningitis in Burkina Faso
Source: PLoS One. 2010 Sep 28;5(9):e13044. doi: 10.1371/journal.pone.0013044 (PMC2946913; doi:10.1371/journal.pone.0013044)
Supplement: Table S4 — Pattern of annual vaccines delivered to region before (1996–2002) and after (2003–2008) IDSR implementation. (0.04 MB DOC) [file pone.0013044.s004.doc]

Table S4

| Time period |  | **GSK vaccine shipped to BF, WHO, MSF, and UNICEF: By vaccine type*** | | | |  | **WHO-ICG vaccine, by type, released to countries in Africa: 1997 to 2007*** | | | | |
| --- | --- | --- | --- | --- | --- | --- | --- | --- | --- | --- | --- |
|  | Year | Bivalent | Trivalent | Tetravalent | Total |  | Bivalent vaccine | Countries | Trivalent vaccine | Countries | **All vaccines** |
| Before IDSR implementation at district level in Burkina Faso | 1990 | 35,000 |  |  | 35,000 |  |  |  |  |  |  |
|  | 1994 | 300,000 |  |  | 300,000 |  |  |  |  |  |  |
|  | 1995 | 100,000 |  |  | 100,000 |  |  |  |  |  |  |
|  | 1996 | 5,229,750 |  |  | 5,229,750 |  |  |  |  |  |  |
|  | 1997 | 1,051,500 |  |  | 1,051,500 |  | 3,105,000 | Not given | 0 |  | **3,105,000** |
|  | 1998 | 1,350,000 |  |  | 1,350,000 |  | 1,550,000 | Chad, Benin, Cameroon, Angola | 0 |  | **1,550,000** |
|  | 1999 | 10,000 |  |  | 10,000 |  | 4,040,000 | Sudan, Guinea Bissau | 0 |  | **4,040,000** |
|  | 2000 | 0 |  |  | 0 |  | 920,000 | Central Africa Republic, Niger, Chad | 0 |  | **920,000** |
|  | 2001 | 1,900,000 |  |  | 1,900,000 |  | 8,144,000 | Burkina Faso, Chad, Ethiopia, Burundi, Cameroon, Niger | 0 |  | **8,144,000** |
|  | 2002 |  |  | 111,320 | 111,320 |  | 200,000 | Ethiopia | 0 |  | **200,000** |
|  |  |  |  |  |  |  |  |  |  |  |  |
| IDSR implementation at district level in Burkina Faso | 2003 |  | 7,453,750 | 25,000 | 7,478,750 |  | 550,000 | Niger | 2,000,000 | Burkina Faso | **2,550,000** |
|  | 2004 |  | 1,500,000 | 260 | 1,500,260 |  | 220,000 | Angola, Central Africa Republic, Chad | 130,000 | Burkina Faso | **350,000** |
|  | 2005 |  |  | 32,060 | 32,060 |  | 200,000 | Chad | 339,250 | Chad, Sudan | **539,250** |
|  | 2006 |  |  | 37,000 | 37,000 |  | 5,036,615 | Burkina Faso, Côte d'Ivoire, Niger, Nigeria, Sudan, Uganda | 1,084,375 | Chad, Guinea, Kenya, Sudan, Uganda | **6,120,990** |
|  | 2007 |  |  | 44,240 | 44,240 |  | 6,924,331 | Burkina Faso, Chad, Democratic Republic of Congo, Sudan, Togo, Uganda | 200,000 | Sudan | **7,124,331** |

*****Acronyms and abbreviations: GSK – GlaxoSmithKline; BF – Burkina Faso (national government); WHO – World Health organization; MSF - Médecins Sans Frontières; UNICEF – United Nations Children’s Fund; ICG - International Consultative Group (from WHO).

**Source:** Doses of vaccine released to countries in Africa by the WHO International Consultative Group, UNICEF, and GlaxoSmithKline Biologicals.
