# Supplementary material for: Modeling the Cost-Effectiveness of the Integrated Disease Surveillance and Response (IDSR) System: Meningitis in Burkina Faso
Source: PLoS One. 2010 Sep 28;5(9):e13044. doi: 10.1371/journal.pone.0013044 (PMC2946913; doi:10.1371/journal.pone.0013044)
Supplement: Table S5 — Annual costs (in 2002 US $) of all public health-related surveillance and IDSR-related activities per category of resources and health structure level in Burkina Faso: 2002 to 2005. (0.07 MB DOC) [file pone.0013044.s005.doc]

Table S5.

| Year | Cost category | **Region** | |  | **District** | |  | **Local** | |
| --- | --- | --- | --- | --- | --- | --- | --- | --- | --- |
|  |  | All health surveillance | Only IDSR |  | All health surveillance | Only IDSR |  | All health surveillance | Only IDSR |
|  |  |  |  |  |  |  |  |  |  |
| 2002 | Personnel | 15,142 | 3,668 |  | 5,865 | 1,287 |  | 1,542 | 411 |
|  | Transportation | 8,850 | 2,990 |  | 8,320 | 1,899 |  | 373 | 155 |
|  | Office | 7,691 | 2,391 |  | 6,648 | 1,099 |  | 474 | 148 |
|  | Media | 1,575 | 212 |  | 257 | 60 |  | 82 | 16 |
|  | Laboratory | 30,662 | 4,510 |  | 7,665 | 1,127 |  | 324 | 48 |
|  | Treatment | 80,808 | 12,952 |  | 20,202 | 3,238 |  | 853 | 137 |
|  | Capital | 5,972 | 2,281 |  | 3,205 | 747 |  | 469 | 205 |
|  | Total | 150,700 | 29,004 |  | 52,162 | 9,458 |  | 4,116 | 1,119 |
|  |  |  |  |  |  |  |  |  |  |
| 2003 | Personnel | 12,971 | 3,007 |  | 6,906 | 1,261 |  | 1,708 | 420 |
|  | Transportation | 18,246 | 5,349 |  | 10,606 | 2,248 |  | 427 | 135 |
|  | Office | 23,472 | 10,405 |  | 8,363 | 1,684 |  | 732 | 182 |
|  | Media | 1,689 | 149 |  | 815 | 254 |  | 254 | 32 |
|  | Laboratory | 23,477 | 4,031 |  | 5,652 | 970 |  | 248 | 43 |
|  | Treatment | 62,131 | 12,423 |  | 14,958 | 2,991 |  | 656 | 131 |
|  | Capital | 11,901 | 4,055 |  | 4,126 | 971 |  | 652 | 179 |
|  | Total | 153,889 | 39,418 |  | 51,425 | 10,379 |  | 4,677 | 1,122 |
|  |  |  |  |  |  |  |  |  |  |
| 2004 | Personnel | 17,609 | 4,165 |  | 9,670 | 2,332 |  | 2,247 | 572 |
|  | Transportation | 8,997 | 3,278 |  | 14,001 | 2,972 |  | 995 | 242 |
|  | Office | 13,256 | 4,989 |  | 8,988 | 2,246 |  | 739 | 198 |
|  | Media | 2,471 | 467 |  | 367 | 69 |  | 359 | 84 |
|  | Laboratory | 31,701 | 5,712 |  | 7,493 | 1,350 |  | 335 | 60 |
|  | Treatment | 41,791 | 13,647 |  | 9,878 | 3,226 |  | 441 | 144 |
|  | Capital | 14,896 | 6,312 |  | 5,717 | 1,102 |  | 665 | 160 |
|  | Total | 130,720 | 38,570 |  | 56,113 | 13,297 |  | 5,782 | 1,461 |
|  |  |  |  |  |  |  |  |  |  |
| 2005 | Personnel | 15,378 | 3,433 |  | 8,500 | 1,864 |  | 1,860 | 509 |
|  | Transportation | 15,967 | 7,467 |  | 9,920 | 1,518 |  | 714 | 132 |
|  | Office | 7,988 | 4,099 |  | 7,423 | 2,197 |  | 2,026 | 216 |
|  | Media | 919 | 123 |  | 668 | 83 |  | 237 | 36 |
|  | Laboratory | 23,259 | 5,874 |  | 5,498 | 1,388 |  | 245 | 62 |
|  | Treatment | 39,126 | 10,541 |  | 9,248 | 2,491 |  | 413 | 111 |
|  | Capital | 12,316 | 5,067 |  | 4,226 | 483 |  | 709 | 192 |
|  | Total | 114,954 | 36,604 |  | 45,483 | 10,024 |  | 6,204 | 1,258 |
