# Supplementary material for: Modeling the Cost-Effectiveness of the Integrated Disease Surveillance and Response (IDSR) System: Meningitis in Burkina Faso
Source: PLoS One. 2010 Sep 28;5(9):e13044. doi: 10.1371/journal.pone.0013044 (PMC2946913; doi:10.1371/journal.pone.0013044)
Supplement: Table S6 — Cost of treating a meningitis-related illness at regional hospital and district health facility levels in Burkina Faso during the 2002 epidemic season. (0.03 MB DOC) [file pone.0013044.s006.doc]

Table S6

|  | **Regional hospital** | |  | **District health facility** | | **Mean** | |
| --- | --- | --- | --- | --- | --- | --- | --- |
|  |  |  |  |  |  |  |  |
|  | FCFA | $US |  | FCFA | $US | FCFA | $US |
|  |  |  |  |  |  |  |  |
| Consultation | 1,050 | 1.51 |  | 100 | 0.14 | 575 | 0.83 |
| Hospitalization | 12,500 | 17.96 |  | 1,000 | 1.44 | 6,750 | 9.70 |
| Drugs | 26,000 | 37.35 |  | 26,000 | 37.35 | 26,000 | 37.35 |
| Specimens test | 10,000 | 14.37 |  | 10,000 | 14.37 | 10,000 | 14.37 |
| Cost per case treated |  |  |  |  |  |  |  |
|  | 49,550 | 71.18 |  | 37,100 | 53.30 | 43,325 | 62.24 |

**Source:** Recherche opérationnelle sur la gratuité de la prise en charge des patients atteints de méningite cérébro-spinale au Burkina Faso. Comité Technique de Pilotage de Lutte contre les Epidémies, Ministère de la Santé, Burkina Faso. Jan. 2003
