# Supplementary material for: Modeling the Cost-Effectiveness of the Integrated Disease Surveillance and Response (IDSR) System: Meningitis in Burkina Faso
Source: PLoS One. 2010 Sep 28;5(9):e13044. doi: 10.1371/journal.pone.0013044 (PMC2946913; doi:10.1371/journal.pone.0013044)
Supplement: Table S7 — Results of statistical analyses of doses of vaccine imported into Burkina Faso from 1996 to 2008: Impact of IDSR on amounts of vaccine imported. (0.03 MB DOC) [file pone.0013044.s007.doc]

Table S7

| Parameter Estimate | Model 1  (n = 13 | Model 2  (n = 12) | Model 3  (n = 13) | Model 4  (n = 12) | Model 5  (n = 12) | Model 6  (n = 11) |
| --- | --- | --- | --- | --- | --- | --- |
|  |  |  |  |  |  |  |
| **Intercept** | 137,788,749 | -507,097,303 | 2,707,969 | -3,206,106 | 17,206,495 | 17,279,405 |
| *Pr > | t |* | *(0.8297)* | *(0.4217)* | *(0.6031)* | *(0.4911)* | *(0.2793)* | *(0.3811)* |
|  |  |  |  |  |  |  |
| **Year** | -67,992 | 254,263 | -1,346 | 1,609 | -8,579 | -8,615 |
| *Pr > | t |* | *(0.8320)* | *(0,4206)* | *(0.6053)* | *(0.4897)* | *(0.2808)* | *(0.3824)* |
|  |  |  |  |  |  |  |
| **IDSR period** | 448,135 | -948,303 | 5,379 | -7,427 | 50,115 | 50,268 |
| *Pr > | t |* | *(0.8522)* | *(0.6593)* | *(0.7822)* | *(0.6416)* | *(0.3635)* | *(0.4205)* |
|  |  |  |  |  |  |  |
| Durbin-Watson* | 1.861 | 2.429 | 1.786 | 2.492 | 2.995 | 2.801 |
|  |  |  |  |  |  |  |

**Notes:** We ran simple linear regression models

Doses imported per year = **ß_o_** (intercept) + **ß_1_** (Year) + **ß_2_** (IDSR)_dummy_ +$\text{ε}$error term

using IDSR implementation period as a dummy variable (Pre-IDSR = 0; Post-IDSR=1).

We run the regression model for the following dependent variables:

Model 1: Total doses imported (including 1996 data)

Model 2: Doses imported (excluding 1996 data)

Model 3: Doses per 100,000 population in whole country (including 1996 data)

Model 4: Doses per 100,000 population in whole country (excluding 1996 data)

Model 5: Doses per 100,000 population in districts where outbreaks occurred (including 1996 data) Model 6: Doses per 100,000 population in districts where outbreaks occurred (excluding 1996 data)

*For each regression model, we calculated the Durbin-Watson statistic to check for autocorrelation. None of the models displayed any statistically significant autocorrelation.
