# Supplementary material for: Modeling the Cost-Effectiveness of the Integrated Disease Surveillance and Response (IDSR) System: Meningitis in Burkina Faso
Source: PLoS One. 2010 Sep 28;5(9):e13044. doi: 10.1371/journal.pone.0013044 (PMC2946913; doi:10.1371/journal.pone.0013044)
Supplement: Table S8 — Pathogens identified by PCR, Latex, and Culture of CSF and serum samples in countries under enhanced surveillance (IDSR) of meningitis in the WHO African region. (0.17 MB DOC) [file pone.0013044.s008.doc]

Table S8. Pathogens identified by PCR, Latex, and Culture of CSF and serum samples in countries under enhanced surveillance (IDSR) of meningitis in the WHO African region

| **Time period of reported outbreaks** | | **Number of pathogen detected** | | | | | |
| --- | --- | --- | --- | --- | --- | --- | --- |
| Year | Country | *Neisseria meningitidis* serogroup A | *Neisseria meningitidis* serogroup W135 | Other *Neisseria meningitidis* serogroup | *Streptococcus pneumoniae* | *Haemophilus iinfluenzae*  type b | Other pathogen |
| 2008 | **Burkina Faso** | **156** | **0** | **0** | **38** | **0** | **3** |
|  | Benin | 8 | 1 | 0 | 75 | 6 | 23 |
|  | RCA | 14 | 0 | 0 | 1 | 0 | 0 |
|  | Ivory Coast | 16 | 1 | 0 | 7 | 2 | 0 |
|  | Mali | 16 | 0 | 0 | 7 | 6 | 8 |
|  | Niger | 833 | 0 | 59 | 73 | 20 | 2 |
|  | Togo | 9 | 5 | 1 | 23 | 6 | 1 |
|  |  |  |  |  |  |  |  |
| 2007 | **Burkina Faso** | **307** | **4** | **0** | **24** | **2** | **0** |
|  | Benin | 32 | 16 | 0 | 83 | 7 | 47 |
|  | Ghana | 5 | 2 | 0 | 15 | 3 | 0 |
|  | Mali | 55 | 5 | 0 | 23 | 7 | 1 |
|  | Niger | 110 | 5 | 9 | 114 | 50 | 2 |
|  | Nigeria | 12 | 1 | 0 | 6 | 0 | 0 |
|  | Chad | 4 | 2 | 0 | 7 | 1 | 0 |
|  | Togo | 84 | 27 | 0 | 25 | 4 | 0 |
|  | DRC* | 2 |  |  |  |  |  |
|  | Sudan* | 23 | 7 |  |  |  |  |
|  | Uganda* | 3 |  |  |  |  |  |
|  |  |  |  |  |  |  |  |
| 2006 | **Burkina Faso** | **237** | **3** | **0** | **26** | **7** | **7** |
|  | Benin | 74 | 10 | 4 | 43 | 7 | 34 |
|  | RCA | 0 | 0 | 1 | 16 | 19 | 0 |
|  | Ethiopia | 1 | 0 | 0 | 0 | 0 | 0 |
|  | Mali | 35 | 0 | 1 | 21 | 17 | 0 |
|  | Niger | 515 | 24 | 600 | 126 | 46 | 22 |
|  | Nigeria | 16 | 0 | 1 | 1 | 0 | 0 |
|  | DRC | 0 | 0 | 2 | - | - | - |
|  | Chad | 41 | 0 | 0 | 11 | 2 | 0 |
|  | Togo | 3 | 0 | 4 | 30 | 4 | 4 |
|  | Ivory Coast* | Id |  |  |  |  |  |
|  | Kenya* |  | Id |  |  |  |  |
|  | Sudan* | 6 | 2 |  |  |  |  |
|  | Uganda* | 3 | 4 |  |  |  |  |
|  |  |  |  |  |  |  |  |
| 2005 | **Burkina Faso** | **15** | **2** | **0** | **47** | **31** | **3** |
|  | Benin | 27 | 3 | 0 | 36 | 3 | 42 |
|  | RCA | 2 | 0 | 0 | 56 | 25 | 0 |
|  | Ivory Coast | 0 | 0 | 0 | 1 | 1 | 0 |
|  | Mali | 6 | 2 | 1 | 26 | 25 | 1 |
|  | Niger | 127 | 24 | 51 | 151 | 37 | 15 |
|  | Nigeria | 0 | 0 | 1 | 0 | 0 | 0 |
|  | DCR | 0 | 0 | 0 | 1 | 0 | 0 |
|  | Chad | 5 | 2 | 0 | 5 | 3 | 0 |
|  | Togo | 0 | 0 | 0 | 0 | 0 | 0 |
|  | Chad* | 9 | 8 |  | 1 | 2 |  |
|  | Eritrea* | Id |  |  |  |  |  |
|  | Sudan* | 3 | 11 |  |  |  |  |
|  |  |  |  |  |  |  |  |
| 2004 | **Burkina Faso** | **128** | **66** | **0** | **177** | **44** |  |
|  | Benin | 23 | 6 | 1 | 47 | 1 |  |
|  | Ivory Coast | 11 | 0 | 1 | 0 | 0 |  |
|  | Ethiopia | 48 | 0 | 2 | 1 | 0 |  |
|  | Ghana | 49 | 2 | 0 | 0 | 0 |  |
|  | Mali | 12 | 4 | 0 | 30 | 16 |  |
|  | Niger | 369 | 32 | 28 | 146 | 47 |  |
|  | Nigeria | ND | ND | ND | ND | ND |  |
|  | DRC | 2 | 0 | 2 | 4 | 6 |  |
|  | Chad | 3 | 0 | 0 | 16 | 8 |  |
|  | Togo | 19 | 1 | 0 | 18 | 2 |  |
|  | Chad* | 7 |  |  |  |  |  |
|  | CAR* | 2 |  |  |  |  |  |
|  |  |  |  |  |  |  |  |
| 2003 | **Burkina Faso** | **264** | **104** |  |  |  |  |
|  | Benin | 8 | 3 |  |  |  |  |
|  | Ghana | 26 | 2 |  |  |  |  |
|  | Mali | 12 | 11 |  |  |  |  |
|  | Niger | 346 | 42 |  |  |  |  |
|  | Nigeria | 5 | 2 |  |  |  |  |
|  | Togo | 35 | 0 |  |  |  |  |
|  |  |  |  |  |  |  |  |
| 2002 | Burkina Faso* | Id | Id |  |  |  |  |
|  | DRC* | Id |  |  |  |  |  |
|  | Ethiopia* | Id |  |  |  |  |  |
|  | Burundi* | 15 | 2 |  |  |  |  |
|  | Tanzania* | 10 |  |  |  |  |  |
|  |  |  |  |  |  |  |  |
| 2001 | Burkina Faso* |  | 14 |  |  |  |  |
|  | Burkina Faso# | 8 | 12 | 2 | 4 |  |  |
|  | Benin* | Id |  |  |  |  |  |
|  | CAR* |  | Id |  |  |  |  |
|  | Chad* | Id |  |  |  |  |  |
|  | Niger* |  | 10 |  |  |  |  |
|  | Niger# | 16 | 12 | 1 | 3 | 2 |  |
|  | Ethiopia* | Id |  |  |  |  |  |
|  | Somalia* | Id |  |  |  |  |  |
|  | Angola* | Id |  |  |  |  |  |
|  |  |  |  |  |  |  |  |
| 2000 | Ethiopia* | 280 |  | 60 |  |  |  |
|  | Rwanda* | Id |  |  |  |  |  |
|  |  |  |  |  |  |  |  |
| 1999 | Sudan** | Id |  |  |  |  |  |
|  |  |  |  |  |  |  |  |
|  |  |  |  |  |  |  |  |
| 1998 |  |  |  |  |  |  |  |
|  |  |  |  |  |  |  |  |
|  |  |  |  |  |  |  |  |
| 1997 | Burkina Faso** |  |  |  |  |  |  |
|  | Ghana** |  |  |  |  |  |  |
|  | Togo** |  |  |  |  |  |  |
|  |  |  |  |  |  |  |  |
| 1996 | Burkina Faso** | Id |  |  |  |  |  |
|  | Mali** | Id |  |  |  |  |  |
|  | Niger** | Id |  |  |  |  |  |
|  | Nigeria** | Id |  |  |  |  |  |
|  | Nigeria^¶^ | 432 |  |  |  | 3 |  |
|  |  |  |  |  |  |  |  |

**Notes:** Numbers of serotype indicate that the serotype was identified in the CSF and serum samples tested and do NOT represent incidence rates by serotype. Id = number of the laboratory confirmed serotype was not reported by the author

Additional data regarding pathogen idnetifcation: During an epidemic in Mali in 1993-1994, 2 of 12 N. meningitidis strains were of serogroup W135 (Ref. Guibiurdenche, M, E.A. Hoiby, J.Y. Riou, F. Varaine, C. Joguet, and D.A. Caugant. 1996. *Epidemics of serogroup A Neisseria meningitidis of subgroup III in Africa*, 1989-1994. Epidemiol. Infect. 116:115-120).

In a 6-year study in Gambia (1990 to 1995), 6 of 16 N. meningitidis isolates were from serogroup W135 (Ref. Kwara, A., R.A. Adegbola, P.T. Corrah, M. Weber, M. Achtman, G. Morelli, D.A. Caugant, and B.M. Greewood. 1998. *Meningitis caused by a serogroup W135 clone of the ET-37 complex of Neisseria meningitidis in West Africa*. Trop. Med. Int. Health 3:742-746).

**Source:** MDSC Meningitis Weekly Bulletin: Weekly feedback bulletin on cerebrospinal meningitis, Countries under Enhanced Surveillance of Meningitis in the WHO African Region, Multi Disease Surveillance Center, Ouagadougou, Burkina Faso ([www.who.int/csr accessed December 2008](http://www.who.int/csr accessed December 2008))

* WHO Disease Outbreak Reported ([www.who.int.csr/don](http://www.who.int.csr/don); accessed December 2008)

** Weekly Epidem. Record 74(9): 65-72 and 71(41): 305-312

# Muhamed-Kheir Taha, I. Parent du Chatelet, M. Schlumberger, I Sanou, S. Djibo, F. de Chabalier, and JM Alonso. 2002. Neisseria meningitis serogroup W135 and A were equally prevalent among meningitis cases occurring at the end of the 2001 epidemics in Burkina Faso and Niger. J. Clinical Microbiology 40(3): 1083-1084

^¶^Mohammed I, A. Nasidi, A.S. Alkali et al. 2000. A severe epidemic of meningococcal meningitis in Nigeria, 1996. Trans. Royal Soc. Trop. Med. Hyg. 94: 265-270
